# Interactome analysis illustrates diverse gene regulatory processes associated with LIN28A in human iPS cell-derived neural progenitor cells

**DOI:** 10.1016/j.isci.2021.103321

**Published:** 2021-10-23

**Authors:** Nam-Kyung Yu, Daniel B. McClatchy, Jolene K. Diedrich, Sarah Romero, Jun-Hyeok Choi, Salvador Martínez-Bartolomé, Claire M. Delahunty, Alysson R. Muotri, John R. Yates

**Affiliations:** 1 Department of Molecular Medicine, The Scripps Research Institute, La Jolla, CA 92037, USA; 2 Department of Pediatrics/Rady Children’s Hospital San Diego, Department of Cellular & Molecular Medicine, School of Medicine, University of California, San Diego, San Diego, CA 92037, USA; 3 Neurobiology Section, Division of Biological Sciences, University of California, San Diego, La Jolla, CA 92093, USA; 4 Stem Cell Program, Center for Academic Research and Training in Anthropogeny (CARTA), Archealization Center (ArchC), Kavli Institute for Brain and Mind, La Jolla, CA 92037, USA

**Keywords:** Gene network, Molecular neuroscience, Omics, Stem cells research

## Abstract

A single protein can be multifaceted depending on the cellular contexts and interacting molecules. LIN28A is an RNA-binding protein that governs developmental timing, cellular proliferation, differentiation, stem cell pluripotency, and metabolism. In addition to its best-known roles in microRNA biogenesis, diverse molecular roles have been recognized. In the nervous system, LIN28A is known to play critical roles in proliferation and differentiation of neural progenitor cells (NPCs). We profiled the endogenous LIN28A-interacting proteins in NPCs differentiated from human induced pluripotent stem (iPS) cells using immunoprecipitation and liquid chromatography-tandem mass spectrometry. We identified over 500 LIN28A-interacting proteins, including 156 RNA-independent interactors. Functions of these proteins span a wide range of gene regulatory processes. Prompted by the interactome data, we revealed that LIN28A may impact the subcellular distribution of its interactors and stress granule formation upon oxidative stress. Overall, our analysis opens multiple avenues for elaborating molecular roles and characteristics of LIN28A.

## Highlights

• IP-MS identified >500 LIN28A-interacting proteins from neural progenitor cells
• Functions of LIN28A-associated proteins span a wide range of gene regulatory processes
• LIN28A impacts subcellular distribution of EIF3D
• LIN28A interferes with SG formation upon oxidative stress

## Graphical abstract

**Figure:**
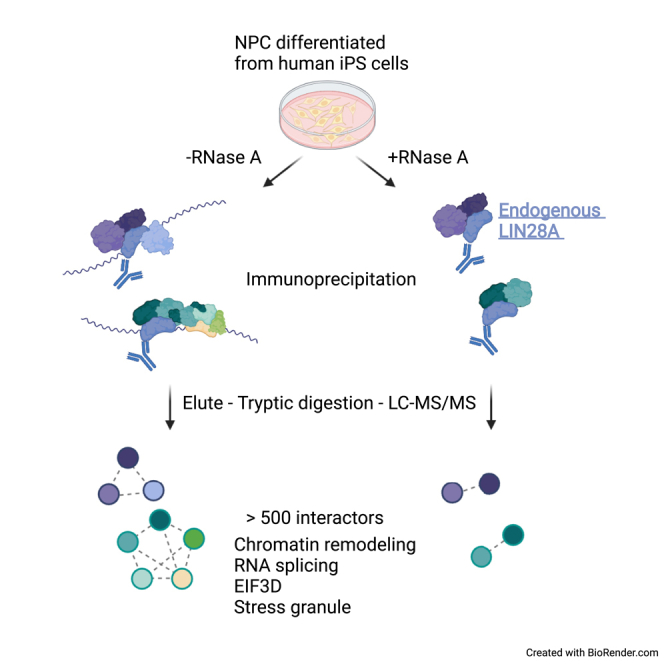

## Introduction

LIN28A was best known as a key negative regulator of let-7 microRNA (miRNA) biogenesis (Heo et al., 2008; Rybak et al., 2008; Viswanathan et al., 2008), but it is also involved in other activities such as regulating translation (Cho et al., 2012; Herrlinger et al., 2019; Jin et al., 2011; Peng et al., 2011; Polesskaya et al., 2007; Qiu et al., 2010; Wilbert et al., 2012; Xu et al., 2009), RNA splicing (Yang et al., 2015a), and DNA methylation (Zeng et al., 2016). Through these divergent molecular pathways, LIN28A plays central roles in a wide range of biological processes such as development (Ambros and Horvitz, 1984; Herrlinger et al., 2019; Moss et al., 1997), pluripotency (Bhattacharya et al., 2018; Shyh-Chang and Daley, 2013), growth (Shinoda et al., 2013), and metabolism (Zhu et al., 2011). LIN28A is emerging as a key oncogene and biomarker for multiple cancers (Jiang and Baltimore, 2016; Li et al., 2012), and its dysregulation has also been implicated in diseases such as diabetes (Thornton and Gregory, 2012) and neurodevelopmental disorders (Kim et al., 2019). LIN28A is one of the four stem cell factors that, in combination, can reprogram somatic cells into pluripotent stem cells (Yu et al., 2007), and it also serves as an enhancer for tissue regeneration (Jo et al., 2016; Shyh-Chang et al., 2013; Wang et al., 2018). Because LIN28A plays such an important role in such a diverse array of processes, a thorough understanding of how it behaves at the molecular level is important for basic biology, pathophysiology, and medicine.

LIN28A impacts multiple steps in neural development, from cell proliferation (Cimadamore et al., 2013; Herrlinger et al., 2019; Romer-Seibert et al., 2019; Yang et al., 2015b) and cell fate determination (Balzer et al., 2010; Herrlinger et al., 2019; Romer-Seibert et al., 2019; Xia et al., 2018) to neurite outgrowth (Jang et al., 2019; Olsson-Carter and Slack, 2010). Typically, LIN28A expression is high at the pluripotent state and declines along with neural differentiation, and both deficiency and overexpression of LIN28A can cause abnormalities. Rett syndrome is a devastating neurodevelopmental disorder, and a recent study reported that induced pluripotent stem (iPS) cell-derived neural progenitor cells (NPCs) from patients with Rett syndrome exhibit aberrant up-regulation of LIN28A and lower glia-to-neuron ratio upon differentiation (Kim et al., 2019). This was phenocopied by LIN28A overexpression in wild-type NPCs, consistent with other studies showing that LIN28A overexpression suppresses gliogenesis while promoting neurogenesis (Balzer et al., 2010; Xia et al., 2018). Although the LIN28A-let-7 axis has been implicated in neurogenesis and NPC proliferation (Cimadamore et al., 2013), it appears that let-7-independent pathways contribute significantly to LIN28A’s effects. When mutant LIN28A lacking the regulatory capability on let-7 was expressed, the effect of LIN28A on gliogenesis was still observed (Balzer et al., 2010). In addition, direct inhibition of let-7 through circular sponge mimicked only a fraction of LIN28A effects on cell fate determination during postnatal neurogenesis (Romer-Seibert et al., 2019). As a let-7-independent mechanism, IGF2-mTOR pathway has been demonstrated to mediate LIN28A’s functions by affecting the translation of target RNAs such as *HMGA2* and *IGF1R* (Xia et al., 2018; Yang et al., 2015b). Recently, it was demonstrated that LIN28A affects NPC proliferation and differentiation by regulating synthesis of proteins related to cell cycles, ribosome biogenesis, and translation (Herrlinger et al., 2019). Beyond these reports, the molecular mechanisms of how LIN28A acts in neural development and related disorders remain largely unknown.

Distinct functions of a protein in different processes are conferred by the context-specific interactions with other molecules. As LIN28A is a well-known RNA binding protein with two tandem Cys-Cys-His-Cys (CCHC)-type zinc finger knuckles and a cold shock domain, the profiles of its binding RNA have been comprehensively analyzed in previous studies (Cho et al., 2012; Hafner et al., 2013; Li et al., 2012; Peng et al., 2011; Wilbert et al., 2012). Thousands of RNA transcripts have been found to be associated with LIN28A, but the effects upon LIN28A perturbation seem to vary (Cho et al., 2012; Tan et al., 2014; Wilbert et al., 2012). This indicates that LIN28A affects its bound RNAs not only by itself but also depending on the interactions with other molecules, most likely proteins (Cho et al., 2012). However, the profiles of LIN28A-interacting proteins are known to a limited extent, particularly in NPC. To our knowledge, only one previous study used NPC-like cells (NE-4C) for surveying LIN28A-interacting proteins (Yang et al., 2015b) and found 40 interactors using GFP-fused Lin28a as the bait. Moreover, regardless of the cell types, most of the identified interactions with LIN28A were not assessed for RNA dependence or assessed but found to require RNA (Heo et al., 2009; Närvä et al., 2012; Parisi et al., 2021; Polesskaya et al., 2007; Yang et al., 2015a). Parsing the RNA-dependent/independent nature of interactions may provide another layer of information to help the mechanistic understanding of LIN28A. To gain a comprehensive molecular view of LIN28A’s roles in the context of neural development, here we analyzed the proteins associated with endogenous LIN28A in human iPS cell-derived NPCs with or without RNase A treatment.

## Results

### LIN28A interactome in human iPS cell-derived NPCs illustrates a wide range of gene regulatory processes associated with LIN28A

**Figure 1:**
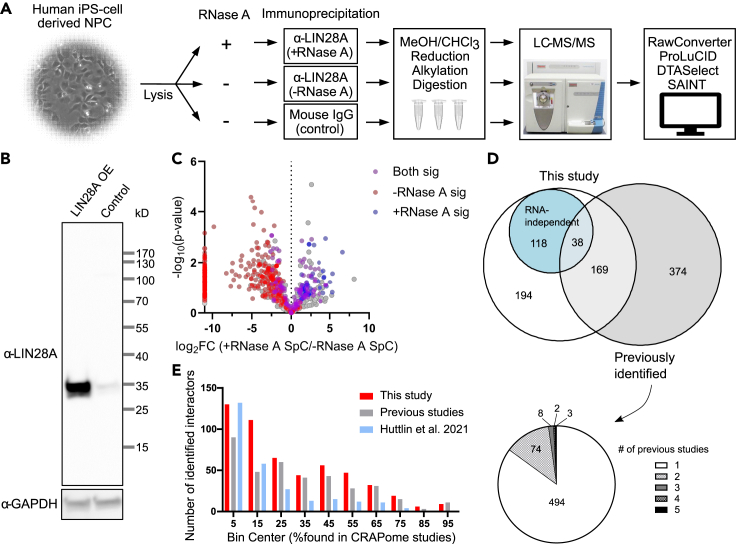
Immunoprecipitation-mass spectrometry (IP-MS) analysis to identify LIN28A interactome (A) Overall workflow of IP-MS used in the present study to identify significant interactors of endogenous LIN28A in human iPS cell-derived NPCs. (B) Western blot assessment of specificity of the LIN28A antibody used in IP-MS. The lysate from LIN28A over-expressing (OE) NPC was loaded next to the same amount of control lysate. The antibody specifically detected LIN28A protein. (C) Volcano plot of the proteins identified in LIN28A IP-MS to compare spectral counts (SpC) in the presence versus absence of RNase A. The y axis represents −log_10_(t test p value). (D) Venn diagram showing the overlap of significant LIN28A interactors identified in this study and those identified in previous studies. Pie chart below represents the number of interactions identified in certain number (1–5) of previous studies. Most of the interactions were identified in only one or two of 23 curated studies (Table S1). (E) Frequency distribution of percentage CRAPome studies that detected the LIN28A interactors in negative controls. LIN28A interactors identified in this study and those in previous studies are represented by interleaved bars in each bin. Recently published large-scale interactome study (Huttlin et al.), in which 273 LIN28A interactors were identified, is separately represented. See also Figure S1.

To identify LIN28A-interacting proteins in human iPS cell-derived NPCs (Chailangkarn et al., 2016), we performed immunoprecipitations (IPs) in biological triplicate using the antibody specific to endogenous LIN28A with or without RNase A treatment and analyzed the eluates by liquid chromatography-coupled mass spectrometry (Figures 1A and S1; Table S1). Proteins identified in the IPs illustrated in Figure 1A are shown in Table S1. LIN28A was detected with the highest abundance among all identified proteins (871 proteins) in +RNase A samples by the average of normalized spectral abundance factor (NSAF) and was ranked as the 24th most abundant protein in −RNase A samples among the 924 identified proteins, indicating that LIN28A was successfully enriched (Figures S1D and S1E). The reason for the lower relative abundance of LIN28A in −RNase A may be the mutual binding of proteins to the same RNA with LIN28A, causing the pull-down of bigger complexes and reducing the relative abundance of LIN28A. The specificity of the antibody was further confirmed by western blotting of NPC lysates (Figure 1B). By comparing with IgG controls using the software tool Significance Analysis of INTeractome (SAINT) (Choi et al., 2011), we identified 457 proteins with false discovery rate < 0.05 in −RNase A (Table S1). In +RNase A, the number of significant interactors was reduced to 156, suggesting that many protein interactions identified in −RNase A were mediated by RNA (Figure 1C). HNRNPA1 was one of the most abundant and significant interactors in −RNase A but was completely abolished in +RNase A (Table S1). This RNA dependency of HNRNPA1-LIN28A interaction recapitulates the result from a previous study (Yang et al., 2015a).

To evaluate the reproducibility and sensitivity of our LIN28A interactome, we surveyed the currently known LIN28A interactors from 23 papers (Amen et al., 2017; Balzer and Moss, 2007; Butland et al., 2014; Chang et al., 2013; Choudhury et al., 2014; Cox et al., 2010; Haenig et al., 2020; Hagan et al., 2009; Haq et al., 2019; Hein et al., 2015; Heo et al., 2009; Huttlin et al., 2021; Jin et al., 2011; Kawahara et al., 2011; Li et al., 2014; Luck et al., 2020; Maier et al., 2015; Mallanna et al., 2010; Marcon et al., 2014; Närvä et al., 2012; Parisi et al., 2021; Polesskaya et al., 2007; Wang et al., 2019), covering more than the BioGRID (Chatr-Aryamontri et al., 2017) and IntAct (Orchard et al., 2014) databases (Table S1). Among 581 previously reported LIN28A interactors, 207 proteins were re-identified in our NPC data. Only 87 of 581 interactions were reproducibly detected in more than one of those 23 studies (Figure 1D), and 52 of those interactions were re-identified in our data (Table S1). Our results newly identify 312 proteins as LIN28A interactors, including most of the RNA-independent interactors (118 of 156) (Figure 1D). To assess whether the identified interactions are due to nonspecific binding of proteins to the beads, we screened our data through CRAPome, the repository of proteins detected in negative controls for affinity purification mass spectrometry (Mellacheruvu et al., 2013) (Figure 1E). Almost half (241 of 519, ∼46.4%) of our interactors have been detected in <20% of CRAPome studies.

**Figure 2:**
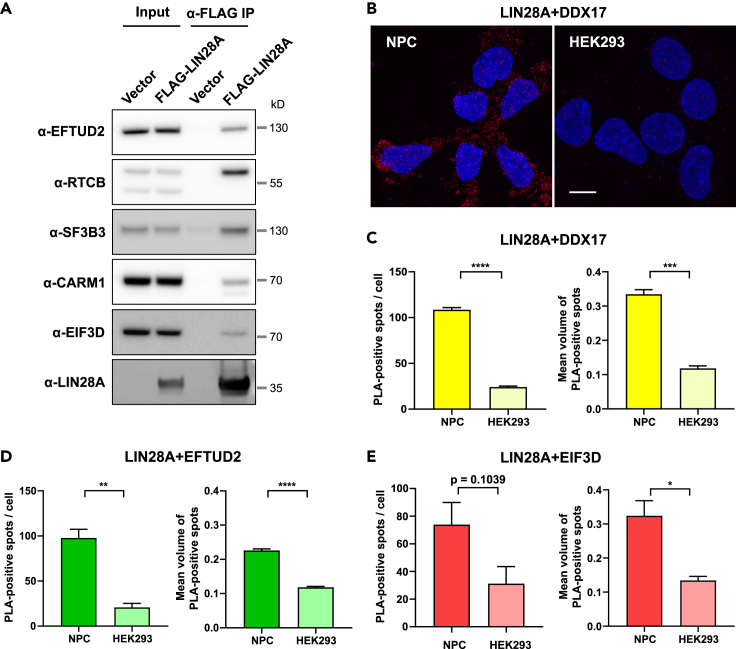
Cross-validation of interactions identified in IP-MS (A) Western blot validation of co-immunoprecipitation in HEK293 cell line. Input lysates (1%) were loaded alongside the IP eluates, and immunoblotting against selected candidates from IP-MS data was performed. (B) Representative images of proximity ligation assays (PLA) to detect the interactions between DDX17 and LIN28A. Scale bar, 10 μm. Red, PLA-positive signals; blue, DAPI. (C–E) Quantification of PLAs to detect interactions of LIN28A with DDX17 (C), EFTUD2 (D), and EIF3D (E). n = 3 different images per group. Statistical significance was calculated by Student’s t test (∗∗∗∗p < 0.0001, ∗∗∗p < 0.001, ∗∗p < 0.01, ∗p < 0.05). Bar graphs represent mean ± SEM. See also Figure S2.

For a cross-validation of our results, we ectopically expressed FLAG-tagged LIN28A in HEK293 cells and purified the associated proteins using anti-FLAG affinity gel. In the protein complexes co-immunopurified with FLAG-LIN28A, we detected proteins such as EFTUD2, RTCB, EIF3D, and CARM1 that are newly identified in this study as well as SF3B3 that was identified in a previous study (Figure 2A). In addition, proximity ligation assays indicated the *in situ* interactions of LIN28A with DDX17, EFTUD2, and EIF3D. Prominently higher level of proximity ligation assay (PLA)-positive spots were observed in NPC than in HEK293 cells that hardly express endogenous LIN28A (Figures 2B–2E). The number of PLA-positive spots appeared significantly higher by each antibody pair (LIN28A-DDX17, LIN28A-EFTUD2, LIN28A-EIF3D) than by negative controls, in which either side of the antibody pairs was replaced with normal IgG (Figure S2).

**Figure 3:**
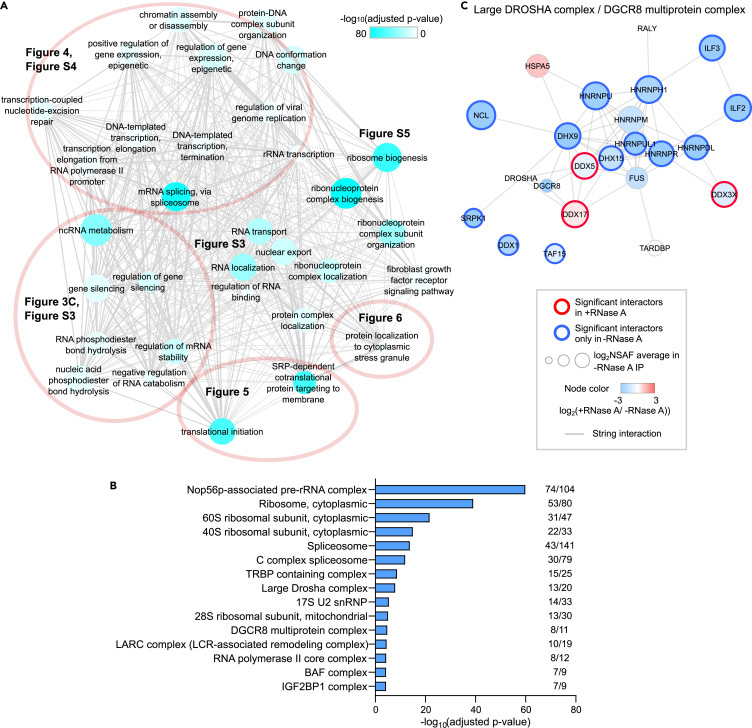
LIN28A interacts with proteins involved in a wide range of gene regulatory processes (A) REVIGO result using GO:BP terms significantly enriched in LIN28A interactors in −RNase A (adjusted p value < 0.001, term size <500 proteins). We comprehensively explored the biological processes associated with LIN28A using the REVIGO result as a map; the relevant figure numbers are represented. (B) The most significantly enriched CORUM complexes in −RNase A (g:Profiler results). Numbers on the right show the number of LIN28A interactors from our results among all the annotated proteins in each complex. (C) LIN28A-associated proteins identified in our data were merged with String networks of proteins annotated in “Large Drosha complex (CORUM:1332)” and “DGCR8 multiprotein complex (CORUM:3082).” Nodes with thick red outlines indicate the interactors that were determined as significant LIN28A interactors in +RNase A, and nodes with thick blue outlines represent significance only in −RNase (A). Node fill color indicates the log_2_(ratio of NSAF (+RNase A IP/−RNase A IP)). Red indicates higher relative abundance in +RNase A, whereas blue means lower relative abundance in +RNase A, suggesting the existence of RNA-dependent interactions. See also Figure S3.

To gain functional insights into the LIN28A-associated molecular network, we performed a gene ontology (GO) enrichment analysis using a software tool g:Profiler (Raudvere et al., 2019). Although RNA-dependent interactions could result from co-binding to the same mRNA, those proteins are likely to be involved in processes occurring at the same time. Indeed, functionally important protein-protein interactions requiring RNA have been reported (Heo et al., 2009; Närvä et al., 2012; Polesskaya et al., 2007; Yang et al., 2015a). Therefore, we surveyed the significant LIN28A interactors in −RNase A IP to first gain a comprehensive overview. In line with LIN28A’s well-known functions, the most enriched terms in GO:BP included RNA metabolic process (GO:0016070, adjusted p value 3.05 × 10^−82^) and translation (GO:0006412, adjusted p value 1.41 × 10^−63^). To highlight more specific functions rather than broad functions such as “gene expression,” we selected significant GO:BP terms of adjusted p value < 0.001 with the term size smaller than 500 proteins for further exploration. The redundant GO terms were clustered using a software tool REVIGO (Supek et al., 2011) (Figure 3A). This overview indicated that LIN28A-associated proteins span a wide range of gene regulatory steps: chromatin organization, transcription, ribosome biogenesis, splicing, RNA transport, mRNA stability, gene silencing, stress granule (SG) localization, and translation. Similarly, various protein complexes retrieved from the CORUM database were significantly enriched (Figure 3B).

As LIN28A is best known for its roles in let-7 miRNA biogenesis, “production of miRNAs involved in gene silencing” (GO:0035196) was significantly enriched in the interactome (adjusted p value for enrichment = 0.000374) (Figure S3A). Protein complexes known to play essential roles in miRNA biogenesis (TRBP-containing complex, Large Drosha complex, DGCR8 multiprotein complex) were overrepresented (Figures 3B, 3C, and S3B). This is in line with previous studies showing that LIN28A is associated with TRBP/Dicer complexes (Amen et al., 2017) and that LIN28A inhibits pri-let-7 processing by Drosha/DGCR8 (Newman et al., 2008; Viswanathan et al., 2008). Drosha and DGCR8 were not significantly detected among LIN28A-associated proteins, suggesting a competitive role of LIN28A against Drosha/DGCR8 from binding the complex components. Most of the interactions were disrupted by RNase A under our conditions, indicating their RNA dependence. LIN28B, DDX5, DDX17, DDX3X, and RPL15 remained as significant interactors in the presence of RNase A, suggesting that their mechanistic relationship is based on protein-protein interactions.

### LIN28A-associated proteins involved in nuclear gene regulatory processes

**Figure 4:**
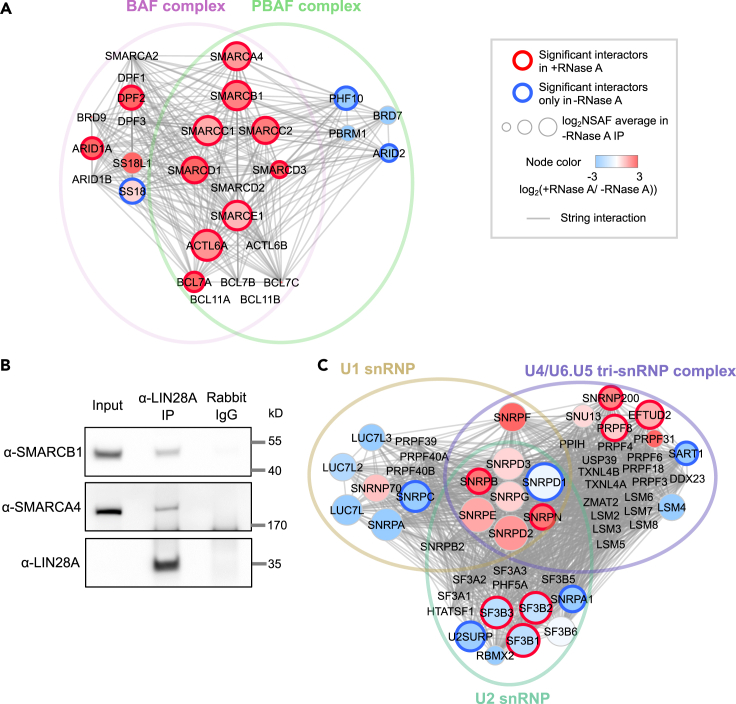
LIN28A-associated proteins involved in nuclear processes: Chromatin organization and splicing (A) String network of BAF/PBAF complex subunits (Hodges et al., 2016) merged with our LIN28A IP-MS data. (B) Immunoblotting of SMARCB1 and SMARCA4 co-immunoprecipitated with LIN28A from NPC lysates in the presence of RNase A. A different antibody (ab63740) was used for IP from the one used for IP-MS (sc-374460) for cross-validation, and normal rabbit IgG was used for the control IP. LIN28A enrichment was so high that LIN28A from the input lysate could not be detected in the same blot with the immunoprecipitated LIN28A. (C) U1 snRNP (GO:0005685), U2 snRNP (GO:0005686), and U4/U6.U5 tri-snRNP (GO:0046540) complex subunits and their interactions, merged with our LIN28A IP-MS data. See also Figures S4 and S5.

Nuclear roles of LIN28A impacting transcription have been rarely explored (Zeng et al., 2016). In our LIN28A interactome, proteins involved in transcription or chromatin organization were overrepresented, implying the existence of unknown roles for LIN28A (Figure 3). Of particular interest, BRG1/BRM-associated factor (BAF) complex subunits were overrepresented in our data (Figure 3B). BAF complex is a subfamily of ATP-dependent chromatin remodeling complexes and known to play crucial roles in neural differentiation, and mutations of its subunits have been frequently found in autistic disorders and cancer (Goodman and Bonni, 2019; Hota and Bruneau, 2016; Mathur and Roberts, 2018; Wilson and Roberts, 2011). Interactions of LIN28A with BAF complex subunits were mostly RNA independent (Figure 4A). We validated the interaction of LIN28A with the BAF complex subunits, SMARCB1 and SMARCA4, by performing western blot after immunoprecipitating LIN28A from NPC lysates in the presence of RNase A (Figure 4B). Interactions specific to PBAF (polybromo- and BAF containing) complex (Hodges et al., 2016), sharing common components with BAF complex, became markedly reduced upon RNase A treatment, suggesting that different types of BAF complexes interact with LIN28A in different manners (Figure 4A). LIN28A interaction with another DNA-binding complex that is involved in DNA replication and repair, RFC complex, was eliminated in the presence of RNase A (Figure S4A). These data imply that LIN28A might be involved in functional effects of cross talk of RNA and DNA-bound complexes (Grossi et al., 2020). As such, LIN28A may affect neural differentiation by interacting with DNA/chromatin-regulatory protein complexes.

LIN28A interactors were prominently enriched with RNA splicing-related proteins (GO:0008380, adjusted p value 1.89 × 10^−81^, Figure 3A). Most eukaryotic mRNAs mature from precursor mRNA (pre-mRNAs) through splicing, during which noncoding introns are excised and protein-coding exons are joined. Regulation of RNA splicing and alternative splicing specific to neural cell types is important for neural development (Porter et al., 2018). Splicing is mediated by the spliceosome, which is a large protein-RNA complex comprising U1, U2, U5, and U4/U6 small nuclear ribonucleoproteins (snRNPs) with additional other proteins. SF3b complex plays a crucial role in pre-mRNA branch site recognition of U2 snRNP during splicing (Gozani et al., 1998). U2 snRNP is assembled on pre-mRNA with U1 snRNP at the initial phase of spliceosome formation, recruiting U4/U6.U5 tri-snRNP (Will and Lührmann, 2011). Intron excision and exon ligation reactions proceed after dissociation of U1 and U4 snRNPs, which requires RNA helicase activity of SNRNP200 (human homolog of Brr2) (Will and Lührmann, 2011). This helicase activity is regulated by EFTUD2 (Bartels et al., 2003) and PRPF8 (Maeder et al., 2009).

A previous study has shown that LIN28A in nuclear extract is associated with HNRNPA1 (Yang et al., 2015a), a key splicing regulatory factor (Jean-Philippe et al., 2013), in breast cancer cells. More than 100 mRNA species were differentially spliced upon LIN28A knockdown, suggesting a role for LIN28A in alternative splicing (Yang et al., 2015a). Yang et al. showed that many of differentially spliced mRNA profiles upon LIN28A knockdown did not overlap with those affected by HNRNPA1 knockdown, suggesting that HNRNPA1-independent mechanisms that mediate the LIN28A’s effects on splicing may exist. In embryonic stem cells and LIN28A-overexpressing HEK293 cells, LIN28A was shown to promote translation of genes encoding splicing-related factors, thereby affecting alternative splicing as a secondary effect (Wilbert et al., 2012). Analyses of LIN28A-bound RNA suggested that major targets of LIN28A seem to be mature mRNAs that have gone through splicing because LIN28A-bound RNA sequences are depleted of intronic regions (Cho et al., 2012; Wilbert et al., 2012). Therefore, whether LIN28A can directly regulate splicing is not yet clear due to the lack of mechanistic understanding.

In our analysis, 112 LIN28A-associated proteins were annotated in RNA splicing (Figure S4B). Upon RNase A treatment, most of those proteins including HNRNPA1 became undetectable or reduced to the control IP level, indicating that those interactions are likely to be mediated by co-bound RNA. Intriguingly, we found that interactions with subunits of U5 snRNP (SNRNP200, EFTUD2, and PRPF8) and U2 snRNP (SF3B1/2/3) remain as significant LIN28A interactors in the presence of RNase A (Figure 4C). In contrast, none of the U1 snRNP-specific components were determined as RNA-independent LIN28A interactors (Figure 4C). Our results support the possibility that LIN28A may directly affect the splicing regulation by protein-protein interactions with U2 snRNP and U5 snRNP components.

### LIN28A impacts subcellular distribution of EIF3D

**Figure 5:**
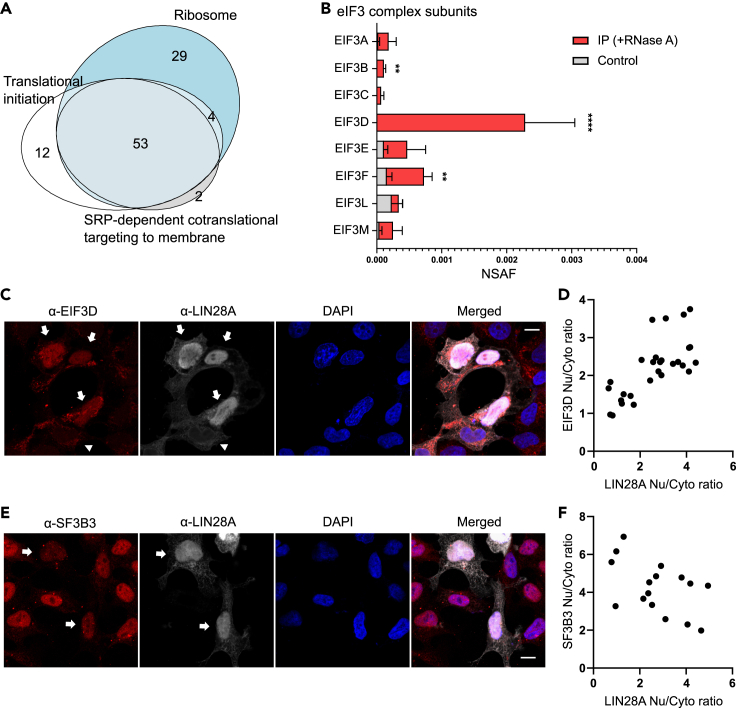
LIN28A impacts subcellular distribution of EIF3D (A) Venn diagram showing the overlaps between the LIN28A-associated proteins annotated in translational initiation (GO:0006413), SRP-dependent cotranslational targeting to membrane (GO:0006614), and ribosome (GO:0005840). (B) Relative abundance of eIF3 subunit proteins co-immunoprecipitated with LIN28A in the presence of RNase A (red bar) and those in the control IP (gray). EIF3G, H, I, K data are omitted as they were detected only once in all the samples. Data are represented as mean ± SEM. ∗∗FDR (false discovery rate) < 0.01, ∗∗∗∗FDR < 0.0001 by SAINT analysis. (C and E) Representative immunofluorescence images of HEK293 cells transfected with LIN28A-expressing plasmids. Scale bar, 10 μm. Arrows indicate cells with intense nuclear LIN28A signals. Arrowheads indicate cells with higher LIN28A signals outside the nucleus. (D) Correlation of nuclear/cytosolic mean intensity ratio of LIN28A and EIF3D. Pearson r = 0.7234, p < 0.0001. (F) Correlation of nuclear/cytosolic mean intensity ratio of LIN28A and SF3B3. Pearson r = −0.5010, p = 0.048. See also Figure S6.

LIN28A is known to be associated with mRNA and polysomes, positively or negatively regulating the translation of mRNA in different contexts (Balzer and Moss, 2007; Cho et al., 2012; Peng et al., 2011; Polesskaya et al., 2007; Qiu et al., 2010; Wilbert et al., 2012; Xu et al., 2009). In line with these previous studies, LIN28A interactors in our analysis were enriched for ribosomal complexes (Figure 3B). The non-ribosomal proteins related with translational initiation (Figure 5A) included EIF3D. eIF3 is the most complex and largest (∼800 kDa) translation initiation factor that plays crucial roles in most forms of translation initiation (des Georges et al., 2015). Three eIF3 subunits (EIF3B, EIF3D, EIF3F) significantly interacted with LIN28A in the presence of RNase A. EIF3D, the peripheral subunit of eIF3 complex thought to be involved in specific translation rather than global translation (Sha et al., 2009), was detected with the highest abundance among the eIF3 subunits and was never detected in the IgG control IP (Figure 5B). The interaction of EIF3D and LIN28A was cross-validated using coimmunoprecipitation and PLA (Figures 2 and S2).

EIF3D has been shown to shuttle between the nucleus and cytosol (Sha et al., 2009; Yen and Chang, 2000). We hypothesized that LIN28A upregulation, observed under pathogenic states such as Rett syndrome (Kim et al., 2019) or cancer (Hamano et al., 2012) or during the trophic response (Amen et al., 2017), may affect subcellular distribution of its interactors by perturbing the protein interaction network. To address this question, we overexpressed LIN28A and surveyed the immunostaining patterns of EIF3D. Interestingly, subcellular distribution of LIN28A itself was heterogeneous among transfected cells (Figures 5C and S6): a subset of cells showed intense nuclear localization of LIN28A, whereas others showed higher cytosolic and perinuclear localization. EIF3D showed prominently higher nuclear intensity in those cells with high level of nuclear LIN28A (Figures 5C and S6A), showing a positive correlation of nuclear/cytosolic ratio of LIN28A and EIF3D (Figure 5D). This effect was specific to EIF3D as this enhanced nuclear signal was not observed for other interactors (SF3B3, EFTUD2) (Figures 5E and S6B). Rather, SF3B3 nuclear intensity tended to be reduced in cells with high nuclear levels of LIN28A (Figure 5F). This demonstrates that LIN28A may impact subcellular distribution of its interactors in different ways. LIN28A may alter the axis of nuclear/cytosolic functions of EIF3D such as nuclear ribosome biogenesis and cytoplasmic translation (Sha et al., 2009).

### LIN28A, associated with stress granule (SG) proteins in the basal state, negatively affects SG assembly upon stress

**Figure 6:**
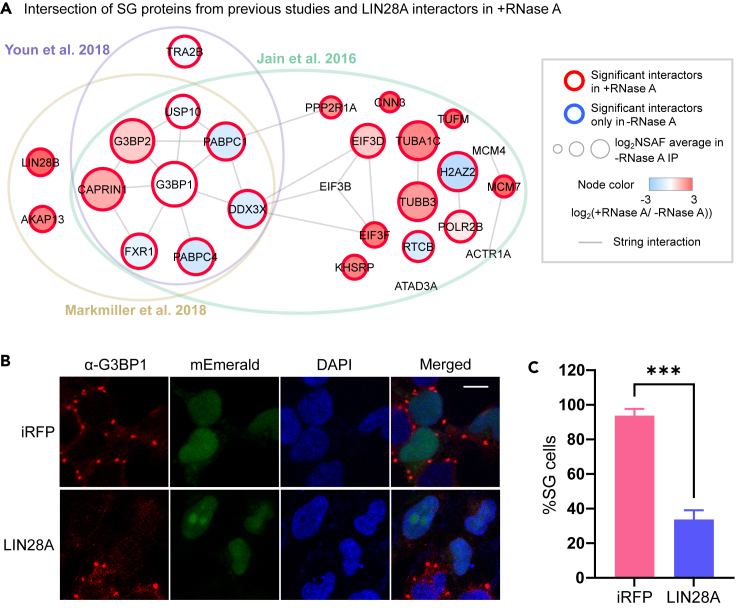
LIN28A impairs stress granule (SG) formation upon stress (A) Intersection of SG proteins from previous studies and RNase A-resistant LIN28A interactors in our study. (B) Immunofluorescence of G3BP1 in infrared fluorescence protein (iRFP) or LIN28A-overexpressing cells with sodium arsenite treatment (0.3 mM, 30 min). mEmerald was expressed as a co-transfection marker. Scale bar, 10 μm. (C) Quantification of percentage SG-positive cells among mEmerald-positive cells (n = 3 plates for each group). At least 35 transfected cells in four images per plate were assessed. Data are represented as mean ± SEM. ∗∗∗p < 0.001, unpaired t test.

SG is a membraneless granule of RNA and RNA-binding proteins, formed under conditions such as heat shock or oxidative stress (Ivanov et al., 2018). It is thought to play important roles in stress-induced gene expression changes and neural differentiation (Jeong et al., 2020). Previous studies have found that LIN28A localizes to SG upon stress (Balzer and Moss, 2007; Markmiller et al., 2018; Palangi et al., 2017). Interestingly, in our LIN28A interactome data, even without stress, SG core proteins, such as G3BP1/2 and CAPRIN1, were highly enriched in our dataset. We compared our data with known SG proteomes defined in three published studies (Jain et al., 2016; Markmiller et al., 2018; Youn et al., 2018). Jain et al. biochemically purified the SG and analyzed the proteins by mass spectrometry, depicting a first SG proteome (Jain et al., 2016). Markmiller et al. defined the SG proteome by proximity-based labeling of G3BP1-interacting proteins in human iPS cell-derived NPCs and non-neural cells (HEK293T) under different types of stress (Markmiller et al., 2018). They found cell-type specific differences in SG proteomes, so we focused only on the NPC SG proteome from that study. Youn et al. systematically performed proximity-based interactome analyses on more than 100 proteins involved in RNA biology and defined core components of SG and P-bodies (Youn et al., 2018). Among 507 SG proteins found in at least one of the three studies, 104 proteins were found as significant LIN28A interactors without RNase A, and 27 interactions were retained in the presence of RNase A (Figure 6A). This indicates that most of the interactions with SG proteins are mediated through co-binding to RNAs, but certain interactions are based on protein-protein interactions. To find out whether LIN28A affects SG formation upon oxidative stress, we overexpressed LIN28A and immunostained the SG marker, G3BP1, after sodium arsenite treatment (Figure 6B). SG formation was markedly impaired in LIN28A-overexpressing cells compared with control cells expressing an infrared fluorescent protein (Figure 6C), suggesting that aberrantly upregulated LIN28A may perturb translation reprogramming through SG assembly.

## Discussion

The mechanisms of LIN28A function are context dependent, and details have been largely elusive. This study provides a comprehensive portrait of the LIN28A interactome, highlighting LIN28A as an all-round player in multiple gene regulatory processes. Our analysis supports and elaborates LIN28A’s previously known roles as well as derives a number of hypotheses regarding the molecular pathways involving LIN28A. The interactions and pathways described may best fit in the context of neural development as we used the human iPS cell-derived NPC, a model previously used to discover the abnormal upregulation of LIN28A in Rett syndrome. We identified more than 500 proteins as significantly associated with LIN28A, including 156 proteins in the presence of RNase A. We pulled down endogenously expressed LIN28A-associated complexes, avoiding potential artifacts that might be caused by over-expressing epitope-tagged LIN28A (Kim et al., 2019).

We identified RNA-independent LIN28A interactors in core splicing machinery as well as those involved in chromatin remodeling, opening a window for studying the nuclear roles of LIN28A and the related gene regulatory events. We found additional LIN28A-associated proteins involved in nuclear processes or RNA metabolism, such as RNA destabilization, polyadenylation, and transport and ribosome biogenesis (Figures 3A, S3C, S3D, and S5), providing a basis for further studies. Our results will help elaborate the mechanistic knowledge of how the pleiotropic LIN28A plays roles in gene regulation.

Translational regulation is crucial for normal brain development, and its dysregulation is known to cause neurodevelopmental disorders such as Rett syndrome and autism-spectrum disorders (Longo and Klann, 2021). EIF3D is known as a peripheral subunit of eIF3 and binds to 5′ cap of mRNA independent of canonical eIF4E-dependent mechanism, especially for expression of specific proteins involved in cell cycle and differentiation (Lee et al., 2015). We found that LIN28A interacts with EIF3D in an RNA-independent manner and that an excess amount of LIN28A in the nucleus increases the nuclear EIF3D. One possible hypothesis is that LIN28A binding to EIF3D might render the nuclear localization signal of EIF3D active. Whether and how the LIN28A-EIF3D interaction impacts translation and ribosome biogenesis particularly during neural development will be interesting to study in the future.

Consistent with previous studies that identified LIN28A as SG components (Balzer and Moss, 2007; Markmiller et al., 2018), we found that LIN28A was associated with SG core proteins (Youn et al., 2018). We determined that the interactions are RNA independent and exist under the unstressed condition. A mechanistic model for SG formation illustrates that pre-existing interactions between SG core proteins form nanoscopic “seeds” at the basal state, which, upon stress, rapidly condensate into microscopic SG by coalescing with neighboring seeds, other proteins, and RNAs (Panas et al., 2016; Youn et al., 2018). Interestingly, we found that LIN28A, which was associated with SG core proteins before stress, markedly impaired the SG formation upon oxidative stress. The mechanisms and physiological impacts of this effect are yet to be studied. We speculate that LIN28A upregulation, which has been reported to be associated with disease states such as Rett syndrome (Kim et al., 2019) or cancer (Balzeau et al., 2017) and trophic responses (Amen et al., 2017), might perturb the molecular interaction network, thereby interfering with the stress response. As well, the excess amount of LIN28A may alter the stress response regulation through EIF3D, which is also a known SG component (Jain et al., 2016). LIN28A may affect the SG-associated translational reprogramming during neural differentiation (Jeong et al., 2020).

We observed that LIN28A preferentially interacts with selective subunits of protein complexes. Whether LIN28A interacts with only a specific subpopulation of the complexes containing the subunits and, if so, what is the structural and functional difference between the LIN28A-associated and non-associated complexes is an appealing question. Those proteins preferentially associated with LIN28A might play a specialized role apart from a canonical complex they belong to. For example, ribosomes are now thought as dynamic and flexible structures rather than static and passive “housekeeping” machineries employed for translation (Genuth and Barna, 2018). Different subunit compositions confer distinct functions and structures in different contexts such as cell types and disease states. Interestingly, LIN28A is associated with specific ribosomal proteins in an RNA-independent mode, and several of these proteins, such as RPL5, RPL10, and RPS27L, have been shown to promote tumorigenesis or cancer (Aspesi and Ellis, 2019; Huang et al., 2013; Xiong et al., 2014). Considering that LIN28A is associated with malignant cancer and poor prognosis (Hamano et al., 2012; Viswanathan et al., 2009; Wang et al., 2015), our data raise the hypothesis that LIN28A might affect the activities of certain ribosomal subunits through protein-protein interactions, leading to a cancer-prone state. Three RNA-independent LIN28A interactors among Drosha/DGCR8 complex subunits are all ATP-dependent RNA helicases with DEAD box (DDX) domain (DDX3X, DDX5, DDX17). They are involved in multiple gene expression processes from transcription to translation similarly to LIN28A (Bourgeois et al., 2016). We verified the LIN28A-DDX17 interaction by PLA, suggesting that they are likely to be direct interactors. It will be interesting to examine whether these multifunctional proteins, binding to broad ranges of RNAs, cooperate to pinpoint specificity of target RNAs and their fates.

Overall, by identifying the protein interactome of endogenous LIN28A in human NPCs differentiated from iPS cells, we provide a molecular basis that may explain how LIN28A plays roles in a wide range of molecular processes, from chromatin organization to translation, in neural differentiation and disorders.

### Limitations of study

Many details remain to be interrogated regarding most of the interactions revealed in this study such as the direct/indirect nature of interactions, the protein domains mediating the interaction, and the functional impact of each interaction.

## STAR★Methods

### Key resources table

**Table:** 

REAGENT or RESOURCE	SOURCE	IDENTIFIER
**Antibodies**		

Anti-LIN28A	Santa Cruz	Cat# sc-374460; RRID:AB_10989468
Anti-LIN28A	Abcam	Cat# Ab-63740; RRID:AB_1310410
Normal mouse IgG	Santa Cruz	Cat# sc-2025; RRID:AB_737182
Mouse IgG2a	Santa Cruz	Cat# sc-3878; RRID:AB_737242
Anti-G3BP1	Proteintech	Cat# 13057-2-AP; RRID:AB_2232034
Anti-EIF3D	Proteintech	Cat# 10219-1-AP; RRID:AB_2096880
Anti-SF3B3	Proteintech	Cat# 19910-1-AP; RRID:AB_10667004
Anti-EFTUD2	Proteintech	Cat# 10208-1-AP; RRID:AB_2095834
Anti-DDX17	Proteintech	Cat# 19910-1-AP; RRID:AB_10667004
Donkey anti-Mouse IgG (H+L) Highly Cross-Adsorbed Secondary Antibody, Alexa Fluor 647	Invitrogen	Cat# A31571; RRID:AB_162542
Alexa Fluor® 594 AffiniPure Donkey Anti-Rabbit IgG (H+L)	Invitrogen	Cat# 711-585-152; RRID:AB_2340621
Normal rabbit IgG	R&D Systems	Cat# AB105C; RRID:AB_354266

**Chemicals, peptides, and recombinant proteins**		

Polyornithine	Sigma	P3655
Laminin	Invitrogen	23017-015
DMEM/F12	Corning	10-092-CV
Gem21	Gemini	400160
NeuroPlex N2	Gemini	400163
bFGF	ThermoFisher	PHG0263
Protein A sepharose	Life Technologies	101041
DMP (dimethyl pimelimidate)	ThermoFisher	21666
cOmplete™, Mini, EDTA-free Protease Inhibitor Cocktail	Sigma	04693159001
PhosSTOP	Sigma	4906845001
ProteaseMax	Promega	V2071
Sequencing grade modified trypsin	Promega	V5111
Lipofectamine 3000	Invitrogen	L3000001
X-tremeGENE™ HP DNA Transfection Reagent	Roche	6366244001
ProLong Diamond with DAPI	Invitrogen	P36971
Opti-MEM	Life Technologies	31985-062

**Critical commercial assays**		

Duolink PLA Technology	Sigma	DUO92101
Duolink In Situ Mounting Medium with DAPI	Sigma	DUO82040

**Deposited data**		

LIN28A immunoprecipitation mass spectrometry data	[Database]: ProteomeXchange consortium through the partner repository MassIVE	PXD015555, MSV000084371

**Experimental models: Cell lines**		

Human: neural progenitor cell	Laboratory of Alysson Muotri	WT83
Human: HEK293	ATCC	CRL-1573

**Recombinant DNA**		

pcDNA3-FLAG.HA	Addgene	10792
pcDNA3-FLAG.LIN28A	Addgene	51371

**Software and algorithms**		

ImageJ	(Schneider et al., 2012)	https://imagej.nih.gov/ij/
RawConverter	(He et al., 2015)	http://fields.scripps.edu/rawconv/
IP2 - Integrated Proteomics Pipeline Ver. 6.7.1	Integrated Proteomics Applications Inc	http://goldfish.scripps.edu/ip2/mainMenu.html
Cytoscape	(Shannon et al., 2003)	https://cytoscape.org/
String	(Szklarczyk et al., 2019)	https://string-db.org/
g:profiler	(Raudvere et al., 2019)	https://biit.cs.ut.ee/gprofiler/gost
SAINT	(Choi et al., 2011)	http://saint-apms.sourceforge.net/Main.html
REVIGO	(Supek et al., 2011)	http://revigo.irb.hr/

### Resource availability

#### Lead contact

Further information and requests for resources and reagents should be directed to and will be fulfilled by the lead contact, John R. Yates III (jyates@scripps.edu).

#### Materials availability

Materials are available upon request.

### Experimental model and subject details

#### Cell culture

Neural progenitor cells (NPCs) differentiated from human induced pluripotent stem (iPS) cells (Chailangkarn et al., 2016) were plated on the dishes coated with poly-ornithine (10 ug/mL, Sigma P3655) in water and then laminin (1:400, Invitrogen 23017-015) in DPBS, each for more than 4 h in 37°C CO_2_ incubator. Cells were maintained in the DMEM/F12 (Corning 10-092-CV) supplemented with Gem21 (1:100, Gemini 400160), NeuroPlex N2 (1:200, Gemini 400163), bFGF (20 ng/mL, PHG0263). Media were changed every other day, and cells were split using Accutase when ∼90% confluency was reached. NPCs were constantly tested for mycoplasma contamination. The Scripps Research Institute Institutional Review Board (TSRI-IRB) approved the protocol. HEK293 cells were maintained in DMEM/10% FBS.

### Method details

#### Plasmid transfection

Plasmids were transfected using X-tremeGENE™ HP DNA Transfection Reagent (Roche). pcDNA3-FLAG-Lin28A (for FLAG-LIN28A overexpression) and pcDNA3-Flag-HA (for vector control) were from Narry Kim lab (Addgene plasmid # 51371) (Heo et al., 2008) and William Sellers lab (Addgene plasmid # 10792), respectively.

#### LIN28A immunoprecipitation

Cells were harvested and centrifuged at 200 ×g, and the cell pellets were stored frozen at −80°C until being processed. For each biological replicate, lysates from six confluent 60 mm plates were pooled and divided into three groups (RNase A+/−, Control). Thereby, all groups, including the negative control, had three biological replicates. Ten μg of LIN28A antibody (Santa Cruz sc-374460) was incubated with 20 μL slurry of Protein A Sepharose (Life Technologies 101041) o/n at 4°C and, washed with 100 mM sodium borate buffer at pH 9.0, and then crosslinked using dimethyl pimelimidate (Thermo 21666, 10 mM) in the borate buffer at room temperature (RT) for 30 min. The crosslinked beads were washed with 200 mM ethanolamine at pH 8.0 and then incubated in the ethanolamine solution at RT for 2 h followed by 2X washing with DPBS and then 3X with cell lysis buffer. Cell pellets were lysed in 20 mM Tris pH 7.5, 200 mM NaCl, 0.5% Triton X-100, 0.5% NP-40 with protease inhibitor cocktails (Sigma 4693159001) and PhosSTOP (Sigma 4906845001) at 4°C and centrifuged at 17K ×g for 30 min. The supernatants were subjected to BCA assay for measuring the protein concentration. One milligram of lysate was precleared using Protein A Sepharose beads pre-incubated with normal mouse IgG. Pre-cleared lysates were incubated with the beads cross-linked with LIN28A antibody or with normal mouse IgG as negative controls at 4°C o/n. For +RNase A samples, RNase A (100 μg/mL) was added and incubated for 30 min at RT before starting the o/n IP. Beads were washed 3 times with 20 mM Tris pH 7.5, 200 mM NaCl and then eluted twice with 5% SDS at 95°C for 10 min.

#### Sample preparation for mass spectrometry

The eluates from the immunoprecipitation were subjected to methanol/chloroform precipitation to extract proteins, and the resulting pellets were dissolved in 4M urea and 0.5% ProteaseMax (Promega) in 50 mM ammonium bicarbonate. After reduction using 5 mM TCEP at 55°C for 20 min in thermo-shaker, samples were alkylated using 10 mM chloroacetamide for 20 min at RT in the dark. After dilution with 50 mM ammonium bicarbonate such that urea becomes ∼2M, trypsin (Promega) 0.5 μg was added and samples were incubated at 37°C for 3 h in thermo-shaker. The tryptic peptides were acidified using formic acid (final 5%), centrifuged at 21K ×g for 10 min, and the supernatants were loaded onto C18 precolumn (250 μm × 2 cm capillary filled with Jupiter 4 μm, 90 Å, Phenomenex).

#### Liquid chromatography–mass spectrometry (LC-MS/MS)

Analysis was performed on Orbitrap Velos Pro mass spectrometer using Xcalibur coupled with an Agilent 1200 G1311 Quaternary HPLC system. In detail, peptides were eluted and separated using an analytical column (100 μm × 15 cm, capillary filled with Jupiter 4 μm, 90 Å, Phenomenex), using a 10 min buffer gradient ranging from 0 to 10% buffer B, followed by a 139 min gradient from 10 to 50% buffer B and 10 min gradient from 50 to 100% buffer B at a flow rate of 250 μL per min utilizing a 1×1000 split flow (buffer A: 0.1% formic acid, 5% acetonitrile; buffer B: 0.1% formic acid, 80% acetonitrile). Data-dependent FTMS acquisition was in positive ion mode for 3 h. A full scan (300–2000m/z) was performed with a resolution of 60,000 followed by top 10 CID-MS2 ion trap scans. Dynamic exclusion was set for 20 s.

#### Bioinformatic analysis and visualization

MS2 spectral data were extracted from the .raw files using RawConverter (He et al., 2015) and searched with IP2 using ProLuCID search engine (Xu et al., 2015) against the human UniProtKB/SwissProt reviewed database (http://www.uniprot.org/, version 2016-05-05). Trypsin was selected as the enzyme and a maximum of 2 missed cleavages were permitted, precursor mass tolerance at 50 ppm and fragment mass tolerance at 600 ppm. Carbamidomethylation of cysteine was defined as a static modification. For DTASelect 2.0 (Cociorva et al., 2006; Tabb et al., 2002), following parameters were applied: -p 1 (one peptide per protein) -y 1 (semitryptic) --pfp 0.01 (protein-level FDR < 1%) -DM 10 (cut-off for precursor mass shift in ppm). The mass spectrometry data have been deposited to the ProteomeXchange consortium (PXD015555) through the partner repository MassIVE (MSV000084371). Significance Analysis of INTeractome (SAINT, v2.0) was used as a statistical tool to determine high-confidence interactions from our data, and spectral counts were used for scoring. Control runs using normal IgG (3 biological replicates) were used as controls, and the proteins with FDR < 0.05 in SAINT analysis were determined as significant interactors. String (v11) (Szklarczyk et al., 2019) was used for network analysis, with Experiments and Databases selected as evidence for the interaction. Interaction data were downloaded and used for visualization in Cytoscape (Shannon et al., 2003). Normalized spectral abundance factors (NSAF) were retrieved from the IP2 results, which equals (the spectral count divided by the length of the protein)/[sum of all (the spectral count divided by the length of the protein) in the run]. Gene ontology enrichment analysis was performed in g: profiler using g:SCS threshold for multiple testing correction. REVIGO was run with allowed similarity 0.5 and *Homo sapiens* as the database.

#### Proximity ligation assay (PLA)

Cells were plated on cover glass-bottomed dish with the density of 30,000 cells/cm^2^. Two days later, cells were fixed with 4% paraformaldehyde in PBS for 15 min at RT and washed with PBS for three times. After permeabilization with 0.5% Triton X-100 in PBS (15 min at RT), cells were washed with PBS and subjected to PLA using Duolink™ PLA Technology (Sigma, DUO92101). After blocking for 1 h at 37°C, primary antibodies at 4 μg/mL [anti-LIN28A (Santa Cruz sc-374460), anti-EIF3D (Proteintech 10219-1-AP), anti-DDX17 (Proteintech 19910-1-AP), anti-LIN28B (Proteintech 24017-1-AP), anti-EFTUD2 (Proteintech 10208-1-AP), mouse IgG2a (Santa Cruz sc-3878), rabbit IgG (R&D Systems AB105C)] were incubated o/n at 4°C, followed by washing with Wash A for 5 min twice at RT and Plus/Minus Probe incubation for 1 h at 37°C. After ligation for 30 min and two washes in Wash A, amplification for 90 min at 37°C, two washes of Wash B for 10 min each, and Wash B 0.01X for 1 min followed at RT. Samples were stored in Duolink™ In Situ Mounting Medium with DAPI (Sigma DUO82040) and imaged with Z-stacks using confocal microscopy (LSM 710) at 100× magnification. PLA-positive spots per image were counted by Object Counter 3D in ImageJ and divided by the cell number.

#### Immunofluorescence

Cell were transfected with plasmids (pAAV-EWB-iRFP670 or pAAV-EWB-LIN28A and pAAV-EWB-mEmerald for co-transfection marker) using Lipofectamine3000 in Opti-MEM following the manufacturer’s protocol. Next day, 0.3 mM NaAsO_2_ (sodium arsenite) was treated for 30 min to induce oxidative stress. After washing with PBS twice, cells were fixed with 4% paraformaldehyde in PBS at RT for 10 min. After washing with PBS three times, cells were permeabilized with 0.5% Triton X-100 in PBS for 15 min at RT, followed by blocking in 3% BSA in 0.1% Triton X-100 for 30 min at RT. Primary antibody was treated at 4°C o/n (anti-G3BP1 1:1000, anti-EIF3D 1:40, anti-LIN28A 1:320, anti-SF3B3 1:150, anti-EFTUD2 1:100). Cells were washed twice with PBT (0.2% TX-100 in PBS) for 15min at RT with gentle shaking and then treated with fluorescent dye-conjugated secondary antibodies (1:500) in blocking solution for 2h at RT. Cells were rinsed twice with PBT and then incubated in PBT with gentle shaking for 15 min. After washing with PBS twice for 15 min, samples were mounted with ProLong Diamond with DAPI. Images were analyzed using ImageJ software. SG positive cells were determined by cells with at least two G3BP1-positive granules.

### Quantification and statistical analysis

Statistical details of each experiments can be found in the method details or figure legends.

Significance Analysis of INTeractome (SAINT, v2.0) was used as a statistical tool to determine high-confidence interactions from IP-MS data. Statistical comparisons of data from immunofluorescence or PLA were performed using GraphPad Prism software v9.1, and the data are represented as mean ± standard error mean (SEM) (∗p < 0.05, ∗∗p < 0.01, ∗∗∗p < 0.001, ∗∗∗∗p < 0.0001).

## Data Availability

• The data have been deposited at ProteomeXchange consortium (PXD015555) through the partner repository MassIVE (MSV000084371) and are publicly available as of the date of publication. Accession numbers are listed in the key resources table. • This paper does not report original code. • Any additional information required to reanalyze the data reported in this paper is available from the lead contact upon request.

## Supplemental information

Document S1. Figures S1–S6

Table S1: Proteins identified in the immunoprecipitations, related to Figure 1A

## References

[bib1] Ambros V., Horvitz H.R. (1984). Heterochronic mutants of the nematode *Caenorhabditis elegans*. Science.

[bib2] Amen A.M., Ruiz-Garzon C.R., Shi J., Subramanian M., Pham D.L., Meffert M.K. (2017). A rapid induction mechanism for Lin28a in trophic responses. Mol. Cel..

[bib3] Aspesi A., Ellis S.R. (2019). Rare ribosomopathies: insights into mechanisms of cancer. Nat. Rev. Cancer.

[bib4] Balzeau J., Menezes M.R., Cao S., Hagan J.P. (2017). The LIN28/let-7 pathway in cancer. Front. Genet..

[bib5] Balzer E., Heine C., Jiang Q., Lee V.M., Moss E.G. (2010). LIN28 alters cell fate succession and acts independently of the let-7 microRNA during neurogliogenesis in vitro. Development.

[bib6] Balzer E., Moss E.G. (2007). Localization of the developmental timing regulator Lin28 to mRNP complexes, P-bodies and stress granules. RNA Biol..

[bib7] Bartels C., Urlaub H., Lührmann R., Fabrizio P. (2003). Mutagenesis suggests several roles of Snu114p in pre-mRNA splicing. J. Biol. Chem..

[bib8] Bhattacharya D., Rothstein M., Azambuja A.P., Simoes-Costa M. (2018). Control of neural crest multipotency by Wnt signaling and the Lin28/let-7 axis. eLife.

[bib9] Bourgeois C.F., Mortreux F., Auboeuf D. (2016). The multiple functions of RNA helicases as drivers and regulators of gene expression. Nat. Rev. Mol. Cell Biol..

[bib10] Butland S.L., Sanders S.S., Schmidt M.E., Riechers S.-P., Lin D.T.S., Martin D.D.O., Vaid K., Graham R.K., Singaraja R.R., Wanker E.E. (2014). The palmitoyl acyltransferase HIP14 shares a high proportion of interactors with Huntingtin: implications for a role in the pathogenesis of Huntington's disease. Hum. Mol. Genet..

[bib11] Chailangkarn T., Trujillo C.A., Freitas B.C., Hrvoj-Mihic B., Herai R.H., Diana X.Y., Brown T.T., Marchetto M.C., Bardy C., McHenry L. (2016). A human neurodevelopmental model for Williams syndrome. Nature.

[bib12] Chang H.-M., Triboulet R., Thornton J.E., Gregory R.I. (2013). A role for the Perlman syndrome exonuclease Dis3l2 in the Lin28-let-7 pathway. Nature.

[bib13] Chatr-Aryamontri A., Oughtred R., Boucher L., Rust J., Chang C., Kolas N.K., O'Donnell L., Oster S., Theesfeld C., Sellam A. (2017). The BioGRID interaction database: 2017 update. Nucleic Acids Res..

[bib14] Cho J., Chang H., Kwon S.C., Kim B., Kim Y., Choe J., Ha M., Kim Y.K., Kim V.N. (2012). LIN28A is a suppressor of ER-associated translation in embryonic stem cells. Cell.

[bib15] Choi H., Larsen B., Lin Z.-Y., Breitkreutz A., Mellacheruvu D., Fermin D., Qin Z.S., Tyers M., Gingras A.-C., Nesvizhskii A.I. (2011). SAINT: probabilistic scoring of affinity purification–mass spectrometry data. Nat. Methods.

[bib16] Choudhury N.R., Nowak J.S., Zuo J., Rappsilber J., Spoel S.H., Michlewski G. (2014). Trim25 is an RNA-specific activator of Lin28a/TuT4-mediated uridylation. Cell Rep..

[bib17] Cimadamore F., Amador-Arjona A., Chen C., Huang C.-T., Terskikh A.V. (2013). SOX2–LIN28/let-7 pathway regulates proliferation and neurogenesis in neural precursors. Proc. Natl. Acad. Sci. U S A.

[bib18] Cociorva D.L., Tabb D., Yates J.R. (2006). Validation of tandem mass spectrometry database search results using DTASelect. Curr. Protoc. Bioinformatics.

[bib19] Cox J.L., Mallanna S.K., Luo X., Rizzino A. (2010). Sox2 uses multiple domains to associate with proteins present in Sox2-protein complexes. PLoS One.

[bib20] des Georges A., Dhote V., Kuhn L., Hellen C.U.T., Pestova T.V., Frank J., Hashem Y. (2015). Structure of mammalian eIF3 in the context of the 43S preinitiation complex. Nature.

[bib21] Genuth N.R., Barna M. (2018). The discovery of ribosome heterogeneity and its implications for gene regulation and organismal life. Mol. Cell.

[bib22] Goodman J.V., Bonni A. (2019). Regulation of neuronal connectivity in the mammalian brain by chromatin remodeling. Curr. Opin. Neurobiol..

[bib23] Gozani O., Potashkin J., Reed R. (1998). A potential role for U2AF-SAP 155 interactions in recruiting U2 snRNP to the branch site. Mol. Cell. Biol..

[bib24] Grossi E., Raimondi I., Goñi E., González J., Marchese F.P., Chapaprieta V., Martín-Subero J.I., Guo S., Huarte M. (2020). A lncRNA-SWI/SNF complex crosstalk controls transcriptional activation at specific promoter regions. Nat. Commun..

[bib25] Haenig C., Atias N., Taylor A.K., Mazza A., Schaefer M.H., Russ J., Riechers S.-P., Jain S., Coughlin M., Fontaine J.-F. (2020). Interactome mapping provides a network of neurodegenerative disease proteins and uncovers widespread protein aggregation in affected brains. Cell Rep..

[bib26] Hafner M., Max K.E., Bandaru P., Morozov P., Gerstberger S., Brown M., Molina H., Tuschl T. (2013). Identification of mRNAs bound and regulated by human LIN28 proteins and molecular requirements for RNA recognition. RNA.

[bib27] Hagan J.P., Piskounova E., Gregory R.I. (2009). Lin28 recruits the TUTase Zcchc11 to inhibit let-7 maturation in mouse embryonic stem cells. Nat. Struct. Mol. Biol..

[bib28] Hamano R., Miyata H., Yamasaki M., Sugimura K., Tanaka K., Kurokawa Y., Nakajima K., Takiguchi S., Fujiwara Y., Mori M. (2012). High expression of Lin28 is associated with tumour aggressiveness and poor prognosis of patients in oesophagus cancer. Br. J. Cancer.

[bib29] Haq S., Das S., Kim D.-H., Chandrasekaran A.P., Hong S.-H., Kim K.-S., Ramakrishna S. (2019). The stability and oncogenic function of LIN28A are regulated by USP28. Biochim. Biophys. Acta Mol. Basis Dis..

[bib30] He L., Diedrich J., Chu Y.-Y., Yates J.R. (2015). Extracting accurate precursor information for tandem mass spectra by RawConverter. Anal. Chem..

[bib31] Hein Marco Y., Hubner Nina C., Poser I., Cox J., Nagaraj N., Toyoda Y., Gak Igor A., Weisswange I., Mansfeld J., Buchholz F. (2015). A human interactome in three quantitative dimensions organized by stoichiometries and abundances. Cell.

[bib32] Heo I., Joo C., Cho J., Ha M., Han J., Kim V.N. (2008). Lin28 mediates the terminal uridylation of let-7 precursor MicroRNA. Mol. Cel..

[bib33] Heo I., Joo C., Kim Y.-K., Ha M., Yoon M.-J., Cho J., Yeom K.-H., Han J., Kim V.N. (2009). TUT4 in concert with Lin28 suppresses microRNA biogenesis through pre-microRNA uridylation. Cell.

[bib34] Herrlinger S., Shao Q., Yang M., Chang Q., Liu Y., Pan X., Yin H., Xie L.-W., Chen J.-F. (2019). Lin28-mediated temporal promotion of protein synthesis is crucial for neural progenitor cell maintenance and brain development in mice. Development.

[bib35] Hodges C., Kirkland J.G., Crabtree G.R. (2016). The many roles of BAF (mSWI/SNF) and PBAF complexes in cancer. Cold Spring Harb. Perspect. Med..

[bib36] Hota S.K., Bruneau B.G. (2016). ATP-dependent chromatin remodeling during mammalian development. Development.

[bib37] Huang C.-J., Yang S.-H., Lee C.-L., Cheng Y.-C., Tai S.-Y., Chien C.-C. (2013). Ribosomal protein S27-like in colorectal cancer: a candidate for predicting prognoses. PLoS One.

[bib38] Huttlin E.L., Bruckner R.J., Navarrete-Perea J., Cannon J.R., Baltier K., Gebreab F., Gygi M.P., Thornock A., Zarraga G., Tam S. (2021). Dual proteome-scale networks reveal cell-specific remodeling of the human interactome. Cell.

[bib39] Ivanov P., Kedersha N., Anderson P. (2018). Stress granules and processing bodies in translational control. Cold Spring Harb. Perspect. Biol..

[bib40] Jain S., Wheeler J.R., Walters R.W., Agrawal A., Barsic A., Parker R. (2016). ATPase-modulated stress granules contain a diverse proteome and substructure. Cell.

[bib41] Jang H.-J., Kim J.Y., Kim S.Y., Cho K.-O. (2019). Persistent Lin28 expression impairs neurite outgrowth and cognitive function in the developing mouse neocortex. Mol. Neurobiol..

[bib42] Jean-Philippe J., Paz S., Caputi M. (2013). hnRNP A1: the Swiss army knife of gene expression. Int. J. Mol. Sci..

[bib43] Jeong S.-G., Ohn T., Jang C.H., Vijayakumar K., Cho G.-W. (2020). The role of stress granules in the neuronal differentiation of stem cells. Mol. Cells.

[bib44] Jiang S., Baltimore D. (2016). RNA-binding protein Lin28 in cancer and immunity. Cancer Lett..

[bib45] Jin J., Jing W., Lei X.-X., Feng C., Peng S., Boris-Lawrie K., Huang Y. (2011). Evidence that Lin28 stimulates translation by recruiting RNA helicase A to polysomes. Nucleic Acids Res..

[bib46] Jo A.-Y., Chang M.-Y., Rhee Y.-H., Song J.-J., Lee S.-H., Oh S.-M., Kim T.-H., Yi S.-H., Park C.-H., Nam J.-W. (2016). LIN28A enhances the therapeutic potential of cultured neural stem cells in a Parkinson’s disease model. Brain.

[bib47] Kawahara H., Okada Y., Imai T., Iwanami A., Mischel P.S., Okano H. (2011). Musashi1 cooperates in abnormal cell lineage protein 28 (Lin28)-mediated let-7 family microRNA biogenesis in early neural differentiation. J. Biol. Chem..

[bib48] Kim J.J., Savas J.N., Miller M.T., Hu X., Carromeu C., Lavallée-Adam M., Freitas B.C., Muotri A.R., Yates J.R., Ghosh A. (2019). Proteomic analyses reveal misregulation of LIN28 expression and delayed timing of glial differentiation in human iPS cells with MECP2 loss-of-function. PLoS One.

[bib49] Lee A.S., Kranzusch P.J., Cate J.H. (2015). eIF3 targets cell-proliferation messenger RNAs for translational activation or repression. Nature.

[bib50] Li N., Zhong X., Lin X., Guo J., Zou L., Tanyi J.L., Shao Z., Liang S., Wang L.-P., Hwang W.-T. (2012). Lin-28 homologue A (LIN28A) promotes cell cycle progression via regulation of cyclin-dependent kinase 2 (CDK2), cyclin D1 (CCND1), and cell division cycle 25 homolog A (CDC25A) expression in cancer. J. Biol. Chem..

[bib51] Li S., Wang L., Fu B., Berman M.A., Diallo A., Dorf M.E. (2014). TRIM65 regulates microRNA activity by ubiquitination of TNRC6. Proc. Natl. Acad. Sci. U S A.

[bib52] Longo F., Klann E. (2021). Reciprocal control of translation and transcription in autism spectrum disorder. EMBO Rep..

[bib53] Luck K., Kim D.-K., Lambourne L., Spirohn K., Begg B.E., Bian W., Brignall R., Cafarelli T., Campos-Laborie F.J., Charloteaux B. (2020). A reference map of the human binary protein interactome. Nature.

[bib54] Maeder C., Kutach A.K., Guthrie C. (2009). ATP-dependent unwinding of U4/U6 snRNAs by the Brr2 helicase requires the C terminus of Prp8. Nat. Struct. Mol. Biol..

[bib55] Maier V.K., Feeney C.M., Taylor J.E., Creech A.L., Qiao J.W., Szanto A., Das P.P., Chevrier N., Cifuentes-Rojas C., Orkin S.H. (2015). Functional proteomic analysis of repressive histone methyltransferase complexes reveals ZNF518B as a G9A regulator. Mol. Cell. Proteomics.

[bib56] Mallanna S.K., Ormsbee B.D., Iacovino M., Gilmore J.M., Cox J.L., Kyba M., Washburn M.P., Rizzino A. (2010). Proteomic analysis of Sox2-associated proteins during early stages of mouse embryonic stem cell differentiation identifies Sox21 as a novel regulator of stem cell fate. Stem Cells.

[bib57] Marcon E., Ni Z., Pu S., Turinsky A.L., Trimble S.S., Olsen J.B., Silverman-Gavrila R., Silverman-Gavrila L., Phanse S., Guo H. (2014). Human-chromatin-related protein interactions identify a demethylase complex required for chromosome segregation. Cell Rep..

[bib58] Markmiller S., Soltanieh S., Server K.L., Mak R., Jin W., Fang M.Y., Luo E.-C., Krach F., Yang D., Sen A. (2018). Context-dependent and disease-specific diversity in protein interactions within stress granules. Cell.

[bib59] Mathur R., Roberts C.W. (2018). SWI/SNF (BAF) complexes: guardians of the epigenome. Annu. Rev. Cancer Biol..

[bib60] Mellacheruvu D., Wright Z., Couzens A.L., Lambert J.-P., St-Denis N.A., Li T., Miteva Y.V., Hauri S., Sardiu M.E., Low T.Y. (2013). The CRAPome: a contaminant repository for affinity purification–mass spectrometry data. Nat. Methods.

[bib61] Moss E.G., Lee R.C., Ambros V. (1997). The cold shock domain protein LIN-28 controls developmental timing in *C. elegans* and is regulated by the lin-4 RNA. Cell.

[bib62] Närvä E., Rahkonen N., Emani M.R., Lund R., Pursiheimo J.P., Nästi J., Autio R., Rasool O., Denessiouk K., Lähdesmäki H. (2012). RNA-Binding protein L1TD1 interacts with LIN28 via RNA and is required for human embryonic stem cell self-renewal and cancer cell proliferation. Stem Cells.

[bib63] Newman M.A., Thomson J.M., Hammond S.M. (2008). Lin-28 interaction with the Let-7 precursor loop mediates regulated microRNA processing. RNA.

[bib64] Olsson-Carter K., Slack F.J. (2010). A developmental timing switch promotes axon outgrowth independent of known guidance receptors. PLoS Genet..

[bib65] Orchard S., Ammari M., Aranda B., Breuza L., Briganti L., Broackes-Carter F., Campbell N.H., Chavali G., Chen C., Del-Toro N. (2014). The MIntAct project—IntAct as a common curation platform for 11 molecular interaction databases. Nucleic Acids Res..

[bib66] Palangi F., Samuel S.M., Thompson I.R., Triggle C.R., Emara M.M. (2017). Effects of oxidative and thermal stresses on stress granule formation in human induced pluripotent stem cells. PLoS One.

[bib67] Panas M.D., Ivanov P., Anderson P. (2016). Mechanistic insights into mammalian stress granule dynamics. J. Cell Biol..

[bib68] Parisi S., Castaldo D., Piscitelli S., D’Ambrosio C., Divisato G., Passaro F., Avolio R., Castellucci A., Gianfico P., Masullo M. (2021). Identification of RNA-binding proteins that partner with Lin28a to regulate Dnmt3a expression. Sci. Rep..

[bib69] Peng S., Chen L.L., Lei X.X., Yang L., Lin H., Carmichael G.G., Huang Y. (2011). Genome-wide studies reveal that Lin28 enhances the translation of genes important for growth and survival of human embryonic stem cells. Stem Cells.

[bib70] Polesskaya A., Cuvellier S., Naguibneva I., Duquet A., Moss E.G., Harel-Bellan A. (2007). Lin-28 binds IGF-2 mRNA and participates in skeletal myogenesis by increasing translation efficiency. Genes Dev..

[bib71] Porter R.S., Jaamour F., Iwase S. (2018). Neuron-specific alternative splicing of transcriptional machineries: implications for neurodevelopmental disorders. Mol. Cell. Neurosci..

[bib72] Qiu C., Ma Y., Wang J., Peng S., Huang Y. (2010). Lin28-mediated post-transcriptional regulation of Oct4 expression in human embryonic stem cells. Nucleic Acids Res..

[bib73] Raudvere U., Kolberg L., Kuzmin I., Arak T., Adler P., Peterson H., Vilo J. (2019). g:profiler: a web server for functional enrichment analysis and conversions of gene lists (2019 update). Nucleic Acids Res..

[bib74] Romer-Seibert J.S., Hartman N.W., Moss E.G. (2019). The RNA-binding protein LIN28 controls progenitor and neuronal cell fate during postnatal neurogenesis. FASEB J..

[bib75] Rybak A., Fuchs H., Smirnova L., Brandt C., Pohl E.E., Nitsch R., Wulczyn F.G. (2008). A feedback loop comprising lin-28 and let-7 controls pre-let-7 maturation during neural stem-cell commitment. Nat. Cell Biol..

[bib76] Schneider C.A., Rasband W.S., Eliceiri K.W. (2012). NIH Image to ImageJ: 25 years of image analysis. Nat. Methods.

[bib77] Sha Z., Brill L.M., Cabrera R., Kleifeld O., Scheliga J.S., Glickman M.H., Chang E.C., Wolf D.A. (2009). The eIF3 interactome reveals the translasome, a supercomplex linking protein synthesis and degradation machineries. Mol. Cell.

[bib78] Shannon P., Markiel A., Ozier O., Baliga N.S., Wang J.T., Ramage D., Amin N., Schwikowski B., Ideker T. (2003). Cytoscape: a software environment for integrated models of biomolecular interaction networks. Genome Res..

[bib79] Shinoda G., Shyh-Chang N., Soysa T.Y.d., Zhu H., Seligson M.T., Shah S.P., Abo-Sido N., Yabuuchi A., Hagan J.P., Gregory R.I. (2013). Fetal deficiency of Lin28 programs life-long aberrations in growth and glucose metabolism. Stem Cells.

[bib80] Shyh-Chang N., Daley G.Q. (2013). Lin28: primal regulator of growth and metabolism in stem cells. Cell Stem Cell.

[bib81] Shyh-Chang N., Zhu H., De Soysa T.Y., Shinoda G., Seligson M.T., Tsanov K.M., Nguyen L., Asara J.M., Cantley L.C., Daley G.Q. (2013). Lin28 enhances tissue repair by reprogramming cellular metabolism. Cell.

[bib82] Supek F., Bošnjak M., Škunca N., Šmuc T. (2011). REVIGO summarizes and visualizes long lists of gene ontology terms. PLoS One.

[bib83] Szklarczyk D., Gable A.L., Lyon D., Junge A., Wyder S., Huerta-Cepas J., Simonovic M., Doncheva N.T., Morris J.H., Bork P. (2019). STRING v11: protein–protein association networks with increased coverage, supporting functional discovery in genome-wide experimental datasets. Nucleic Acids Res..

[bib84] Tabb D.L., McDonald W.H., Yates J.R. (2002). DTASelect and contrast: tools for assembling and comparing protein identifications from shotgun proteomics. J. Proteome Res..

[bib85] Tan S.M., Altschuler G., Zhao T.Y., Ang H.S., Yang H., Lim B., Vardy L., Hide W., Thomson A.M., Lareu R.R. (2014). Divergent LIN28-mRNA associations result in translational suppression upon the initiation of differentiation. Nucleic Acids Res..

[bib86] Thornton J.E., Gregory R.I. (2012). How does Lin28 let-7 control development and disease?. Trends Cell Biol..

[bib87] Viswanathan S.R., Daley G.Q., Gregory R.I. (2008). Selective blockade of microRNA processing by Lin28. Science.

[bib88] Viswanathan S.R., Powers J.T., Einhorn W., Hoshida Y., Ng T.L., Toffanin S., O'Sullivan M., Lu J., Phillips L.A., Lockhart V.L. (2009). Lin28 promotes transformation and is associated with advanced human malignancies. Nat. Genet..

[bib89] Wang S., Chim B., Su Y., Khil P., Wong M., Wang X., Foroushani A., Smith P.T., Liu X., Li R. (2019). Enhancement of LIN28B-induced hematopoietic reprogramming by IGF2BP3. Genes Dev..

[bib90] Wang T., Wang G., Hao D., Liu X., Wang D., Ning N., Li X. (2015). Aberrant regulation of the LIN28A/LIN28B and let-7 loop in human malignant tumors and its effects on the hallmarks of cancer. Mol. Cancer.

[bib91] Wang X.-W., Li Q., Liu C.-M., Hall P.A., Jiang J.-J., Katchis C.D., Kang S., Dong B.C., Li S., Zhou F.-Q. (2018). Lin28 signaling supports mammalian PNS and CNS axon regeneration. Cell Rep..

[bib92] Wilbert M.L., Huelga S.C., Kapeli K., Stark T.J., Liang T.Y., Chen S.X., Yan B.Y., Nathanson J.L., Hutt K.R., Lovci M.T. (2012). LIN28 binds messenger RNAs at GGAGA motifs and regulates splicing factor abundance. Mol. Cel..

[bib93] Will C.L., Lührmann R. (2011). Spliceosome structure and function. Cold Spring Harb. Perspect. Biol..

[bib94] Wilson B.G., Roberts C.W. (2011). SWI/SNF nucleosome remodellers and cancer. Nat. Rev. Cancer.

[bib95] Xia X., Teotia P., Ahmad I. (2018). Lin28a regulates neurogliogenesis in mammalian retina through the Igf signaling. Dev. Biol..

[bib96] Xiong X., Zhao Y., Tang F., Wei D., Thomas D., Wang X., Liu Y., Zheng P., Sun Y. (2014). Ribosomal protein S27-like is a physiological regulator of p53 that suppresses genomic instability and tumorigenesis. eLife.

[bib97] Xu B., Zhang K., Huang Y. (2009). Lin28 modulates cell growth and associates with a subset of cell cycle regulator mRNAs in mouse embryonic stem cells. RNA.

[bib98] Xu T., Park S., Venable J., Wohlschlegel J., Diedrich J., Cociorva D., Lu B., Liao L., Hewel J., Han X. (2015). ProLuCID: an improved SEQUEST-like algorithm with enhanced sensitivity and specificity. J. Proteomics.

[bib99] Yang J., Bennett B.D., Luo S., Inoue K., Grimm S.A., Schroth G.P., Bushel P.R., Kinyamu H.K., Archer T.K. (2015). LIN28A modulates splicing and gene expression programs in breast cancer cells. Mol. Cell. Biol..

[bib100] Yang M., Yang S.-L., Herrlinger S., Liang C., Dzieciatkowska M., Hansen K.C., Desai R., Nagy A., Niswander L., Moss E.G. (2015). Lin28 promotes the proliferative capacity of neural progenitor cells in brain development. Development.

[bib101] Yen H.C., Chang E.C. (2000). Yin6, a fission yeast Int6 homolog, complexes with Moe1 and plays a role in chromosome segregation. Proc. Natl. Acad. Sci. U S A.

[bib102] Youn J.-Y., Dunham W.H., Hong S.J., Knight J.D., Bashkurov M., Chen G.I., Bagci H., Rathod B., MacLeod G., Eng S.W. (2018). High-density proximity mapping reveals the subcellular organization of mRNA-associated granules and bodies. Mol. Cell.

[bib103] Yu J., Vodyanik M.A., Smuga-Otto K., Antosiewicz-Bourget J., Frane J.L., Tian S., Nie J., Jonsdottir G.A., Ruotti V., Stewart R. (2007). Induced pluripotent stem cell lines derived from human somatic cells. Science.

[bib104] Zeng Y., Yao B., Shin J., Lin L., Kim N., Song Q., Liu S., Su Y., Guo J.U., Huang L. (2016). Lin28A binds active promoters and recruits Tet1 to regulate gene expression. Mol. Cell.

[bib105] Zhu H., Shyh-Chang N., Segrè A.V., Shinoda G., Shah S.P., Einhorn W.S., Takeuchi A., Engreitz J.M., Hagan J.P., Kharas M.G. (2011). The Lin28/let-7 axis regulates glucose metabolism. Cell.

