# Supplementary material for: Interactome analysis illustrates diverse gene regulatory processes associated with LIN28A in human iPS cell-derived neural progenitor cells
Source: iScience. 2021 Oct 23;24(11):103321. doi: 10.1016/j.isci.2021.103321 (PMC8593586; doi:10.1016/j.isci.2021.103321)
Supplement: Document S1. Figures S1–S6 [file mmc1.pdf]

iScience, Volume *24*

Supplemental information

Interactome analysis illustrates diverse gene regulatory processes associated with LIN28A in human iPS cell-derived neural progenitor cells

Nam-Kyung Yu, Daniel B. McClatchy, Jolene K. Diedrich, Sarah Romero, Jun-Hyeok Choi, Salvador Martínez-Bartolomé, Claire M. Delahunty, Alysson R. Muotri, and John R. Yates III

A

	-RNase A_1	-RNase A_2	-RNase A_3	+RNase A_1	+RNase A_2	+RNase A_3	Control_1	Control_2	Control_3
-RNase A_1	1								
-RNase A_2	0.929543	1							
-RNase A_3	0.917908	0.875217	1						
+RNase A_1	0.508224	0.480373	0.518712	1					
+RNase A_2	0.384021	0.405303	0.385044	0.760861	1				
+RNase A_3	0.424611	0.43202	0.446629	0.878987	0.765866	1			
Control_1	0.341437	0.337591	0.331979	0.437674	0.428878	0.236299	1		
Control_2	0.367121	0.335726	0.328643	0.377324	0.376772	0.232355	0.696707	1	
Control_3	0.335171	0.37016	0.352738	0.486265	0.538455	0.331946	0.771513	0.725815	1

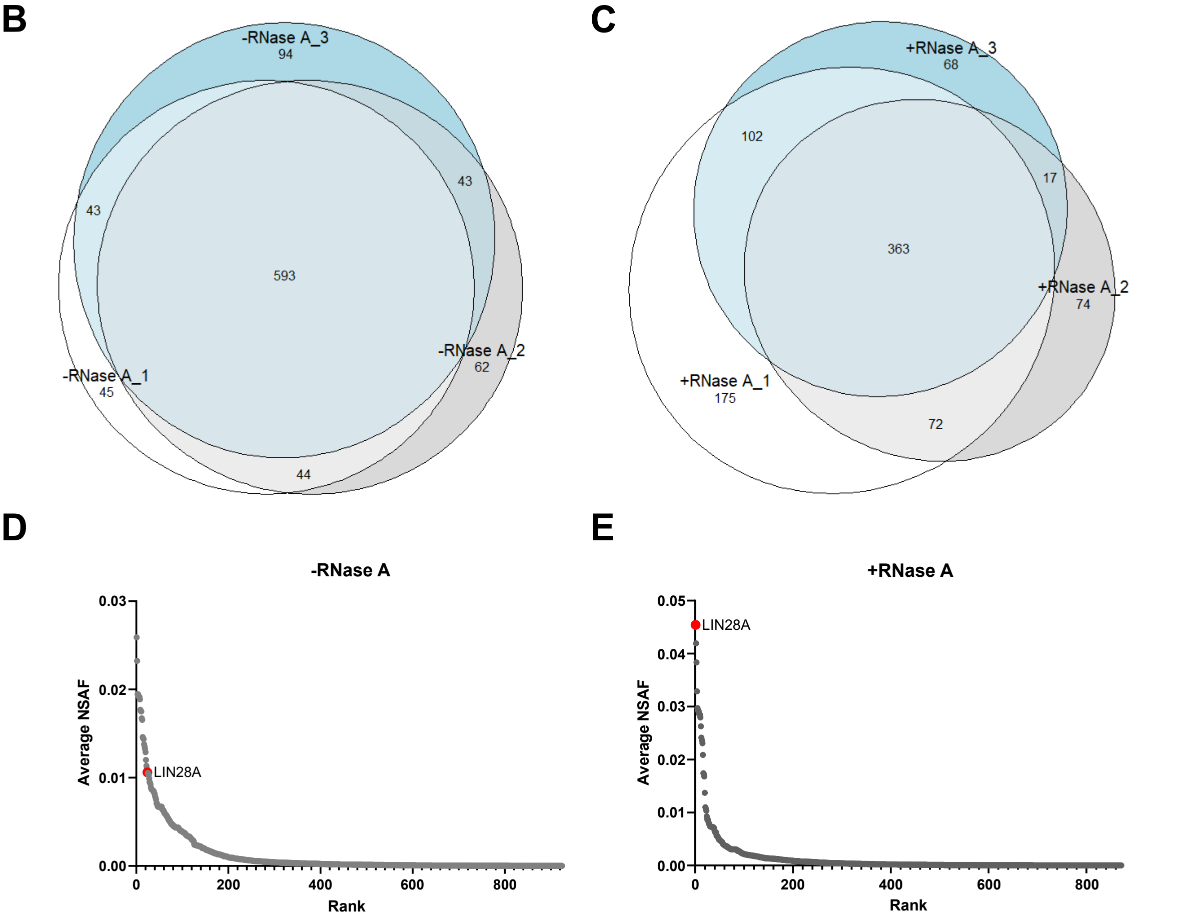

Figure S1. Assessment of LIN28A immunoprecipitation data (Related to Figure 1)
(A) Correlation matrix of normalized spectral abundance factor (NSAF) values of proteins identified in all samples for each pair of immunoprecipitated samples.
(B, C) Overlap of identified proteins between biological replicates.
(D, E) Rank plots of average NSAF of identified proteins in -RNase A group (D) and +RNase A group (E).

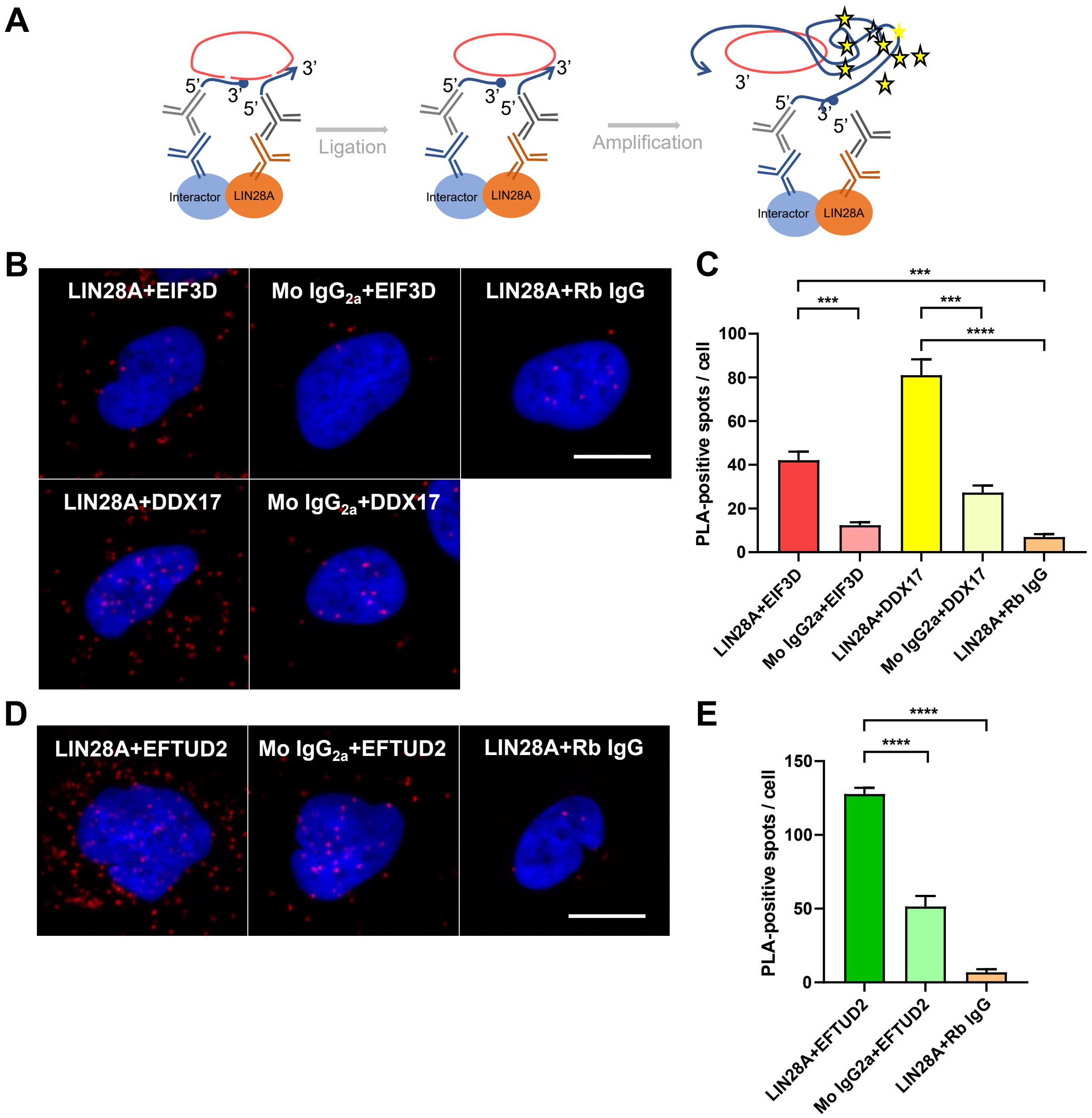

Figure S2. Proximity ligation assay (PLA) to verify the interaction of LIN28A and selected proteins (Related to Figure 2) (A) Scheme of PLA to examine protein-protein interactions. (B, D) Representative images from PLA to verify LIN28A-EIF3D or LIN28A-DDX17 interactions (B) and LIN28A-EFTUD2 interaction (D). (C, E) Quantification of PLA-positive spots per cell in (B) and (D), respectively (n=3 images per each group, each image contained 3-7 cells). Bar graphs are represented as mean ± SEM. Scale bars, 10 µm. One way ANOVA, Tukey's multiple comparisons test, ***p < 0.001, ****p < 0.0001.

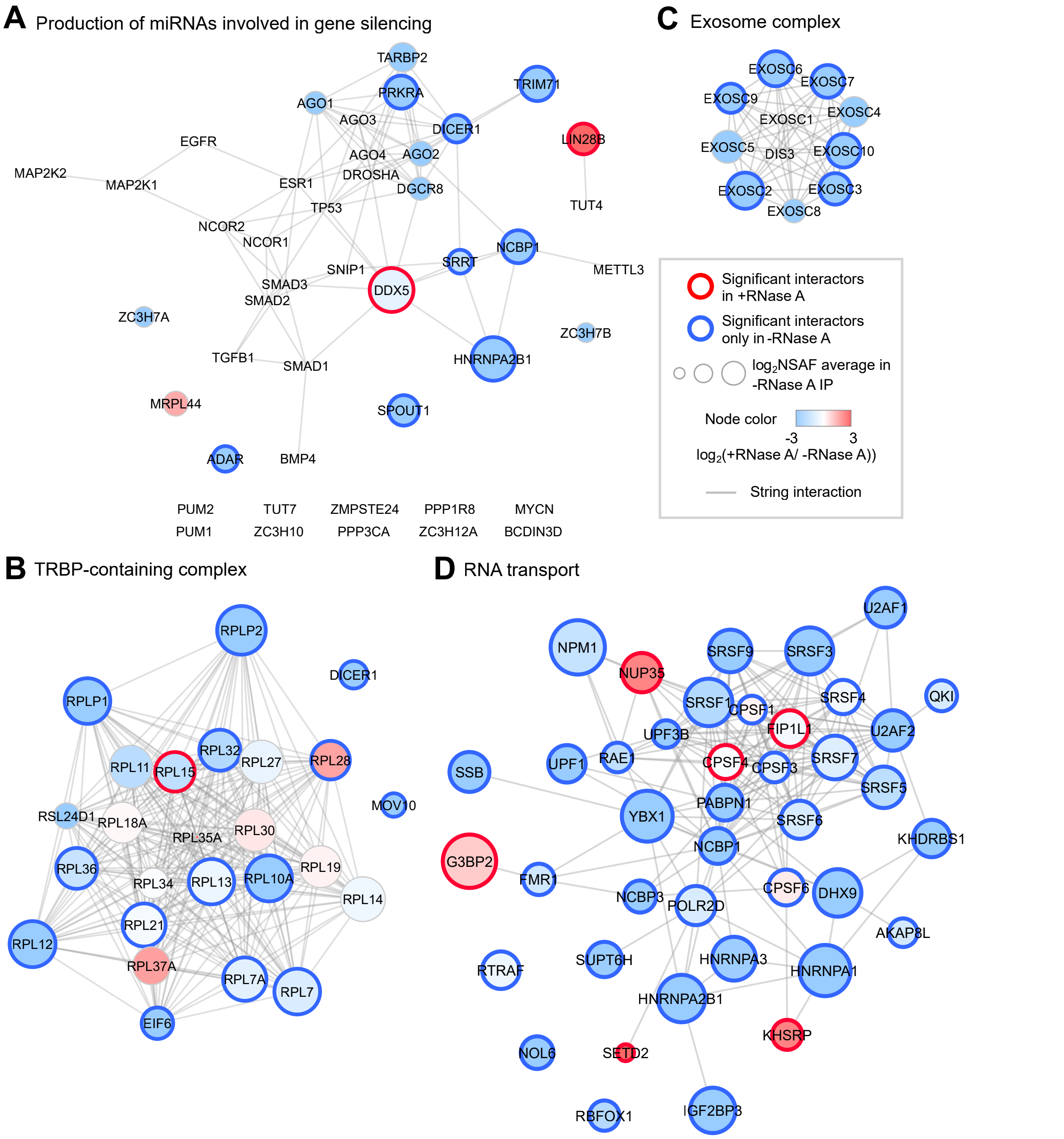

Figure S3. LIN28A is associated with proteins involved in RNA metabolism and transport (Related to Figure 3). LIN28A-associated proteins identified in our data were merged upon String networks of proteins involved in miRNA biogenesis. (A) Proteins annotated in “Production of miRNAs involved in gene silencing (GO:0035196)”. (B) Proteins annotated in “TRBP-containing complex (CORUM:5380)” (C) Proteins annotated in “Exosome complex (CORUM:789)”. (D) Significant LIN28A interactors annotated in “RNA transport (GO:0050658)”. Note that CPSF1, 3, 4 and FIP1L1 belong to cleavage and polyadenylation factor complex.

A RFC complex

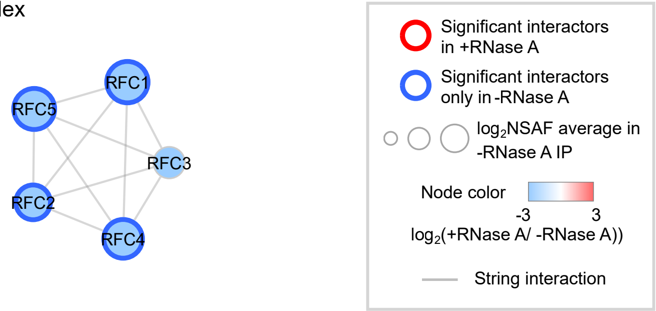

B RNA splicing

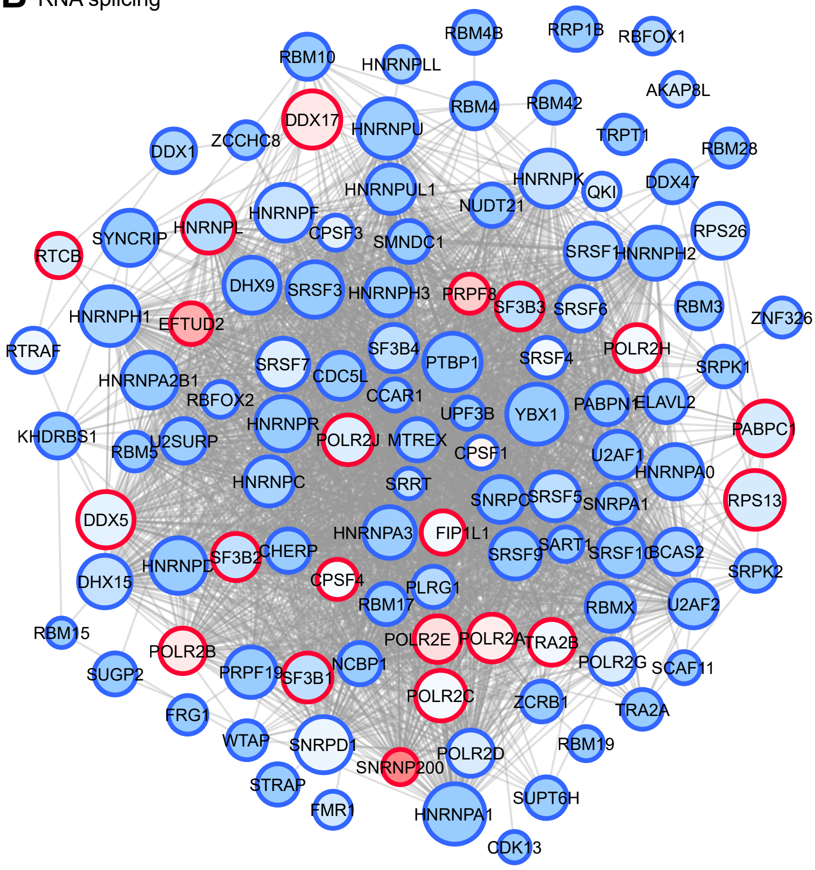

Figure S4. LIN28A is associated with proteins involved in nuclear gene regulatory processes (Related to Figure 4). (A) RFC complex (CORUM:279) (B) Significant LIN28A interactors annotated in “RNA splicing (GO:0008380)”.

A Ribosome biogenesis

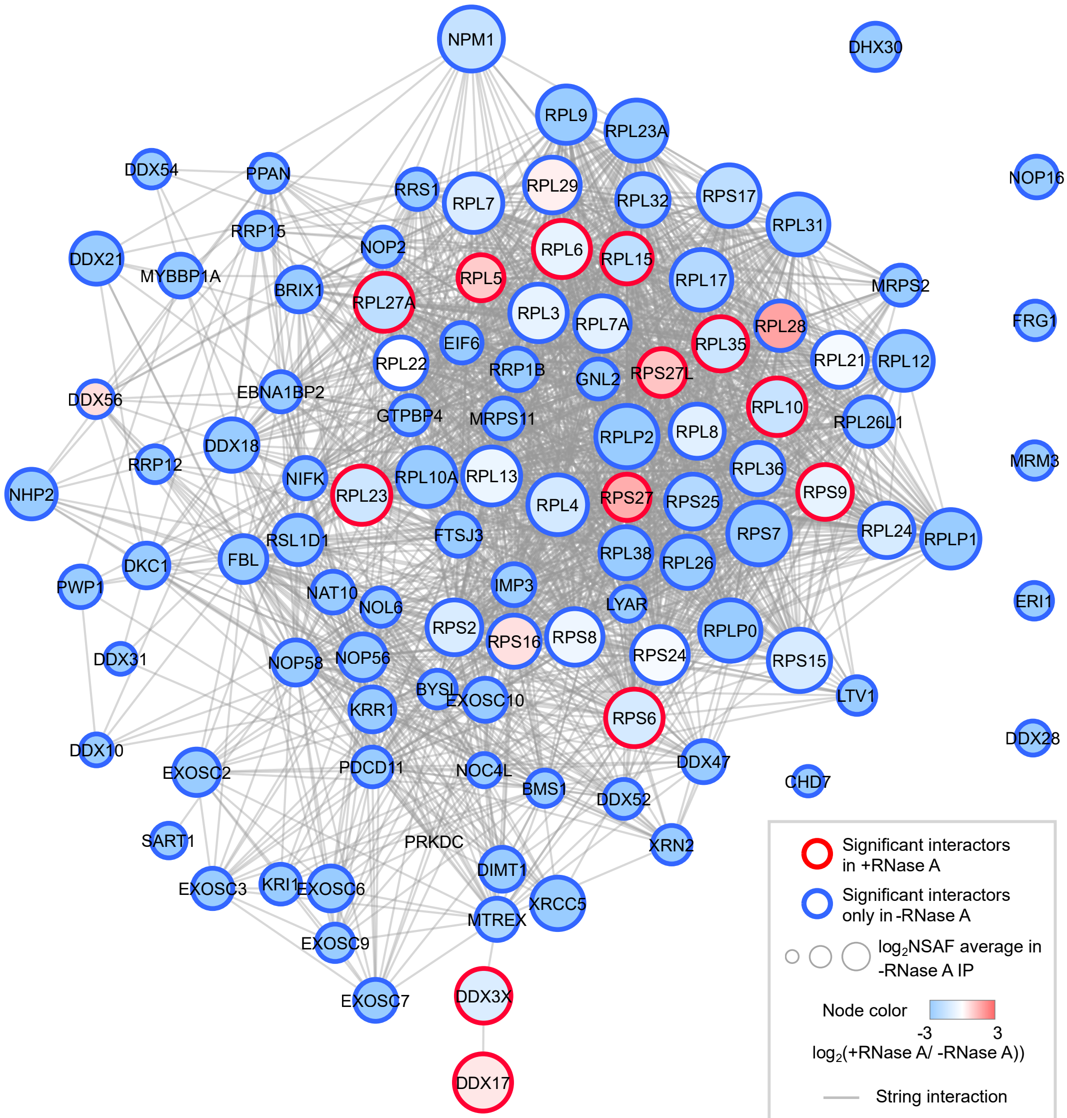

Figure S5. LIN28A is associated with proteins involved in ribosome biogenesis (Related to Figure 4)
Significant LIN28A interactors annotated in “ribosome biogenesis (GO:0042254)”.

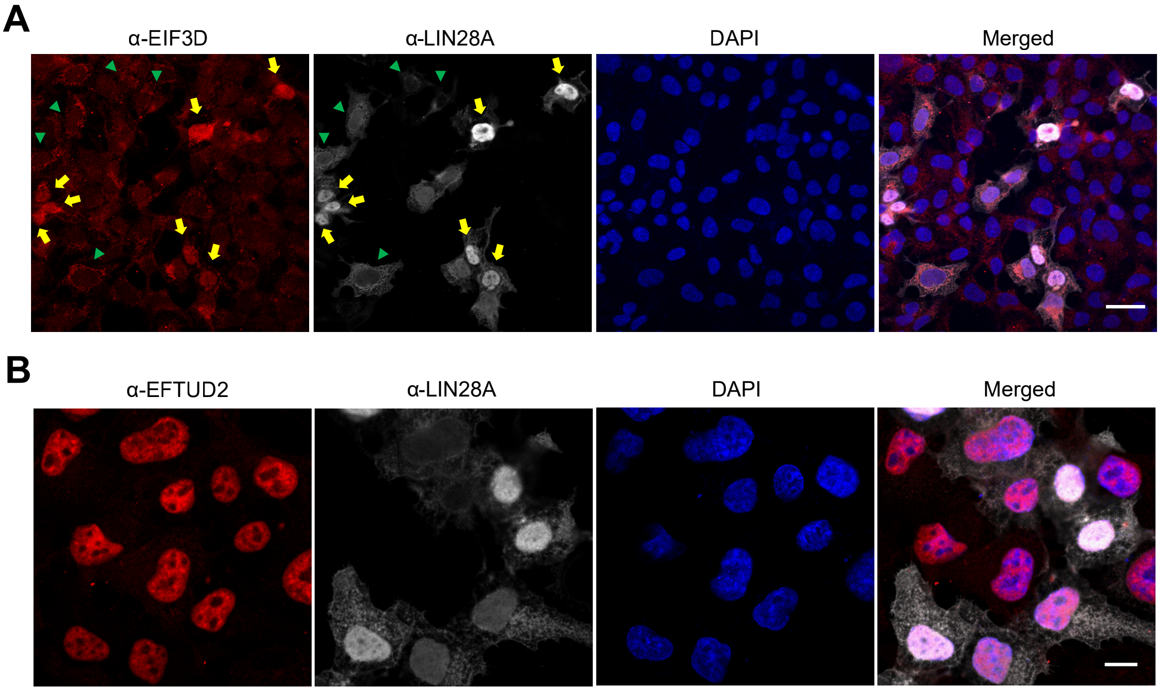

Figure S6. LIN28A impacts subcellular distribution of EIF3D (Related to Figure 5) (A-B) Representative immunofluorescence images of HEK293 cells transfected with LIN28A-expressing plasmids. Yellow arrows, cells with intense nuclear LIN28A signals. Green arrowheads, cells with low nuclear LIN28A signals. Scale bars, 30 μm (A) and 10 μm (B).
